# Efficacy of Initial Antiretroviral Therapy for HIV-1 Infection in Adults: A Systematic Review and Meta-Analysis of 114 Studies with up to 144 Weeks' Follow-Up

**DOI:** 10.1371/journal.pone.0097482

**Published:** 2014-05-15

**Authors:** Frederick J. Lee, Janaki Amin, Andrew Carr

**Affiliations:** 1 Clinical Research Program, St. Vincent's Centre for Applied Medical Research, Sydney, Australia; 2 The Kirby Institute, University of New South Wales, Sydney, Australia; 3 HIV, Immunology and Infectious Diseases Unit, St. Vincent's Hospital, Sydney, Australia; McGill University AIDS Centre, Canada

## Abstract

**Background:**

A comprehensive assessment of initial HIV-1 treatment success may inform study design and treatment guidelines.

**Methods:**

Group-based, systematic review and meta-analysis of initial antiretroviral therapy studies, in adults, of ≥48 weeks duration, reported through December 31, 2012. Size-weighted, intention-to-treat efficacy was calculated. Parameters of study design/eligibility, participant and treatment characteristics were abstracted. Multivariable, random effects, linear regression models with backwards, stepwise selection were then used to identify variables associated with efficacy.

**Outcome Measures:**

Antiviral efficacy (undetectable plasma viral load) and premature cessation of therapy.

**Results:**

114 studies were included (216 treatment groups; 40,124 participants; mean CD4 count 248 cells/µL [SD 81]; mean HIV-1 plasma viral load log_10_ 4.9 [SD 0.2]). Mean efficacy across all groups was 60% (SD 16) over a mean 82 weeks' follow-up (SD 38). Efficacy declined over time: 66% (SD 16) at 48 weeks, 60% (SD 16) at 96 weeks, 52% (SD 18) at 144 weeks. The most common reason for treatment cessation was participant decision (11%, SD 6.6). Efficacy was higher with ‘Preferred’ than ‘Alternative’ regimens (as defined by 2013 United States antiretroviral guidelines): 75% vs. 65%, respectively, difference 10%; 95%CI 7.6 to 15.4; *p*<0.001. In 98 groups (45%) reporting efficacy stratified by pre-treatment viral load (< or ≥100,000 copies/mL), efficacy was greater for the lower stratum (70% vs. 62%, respectively, difference 8.4%; 95%CI 6.0 to 10.9; *p*<0.001). This difference persisted within ‘Preferred’ regimens. Greatest efficacy was associated with use of tenofovir-emtricitabine (vs. other nucleoside analogue backbones) and integrase strand transfer inhibitors (vs. other third drug classes).

**Conclusion:**

Initial antiretroviral treatments for HIV-1 to date appear to have suboptimal long-term efficacy, but are more effective when commenced at plasma viral loads <100,000 copies/mL. Rising viral load should be considered an indication for starting treatment. Integrase inhibitors offer a treatment advantage (vs. other third drug classes).

## Introduction

Combination antiretroviral therapy (cART) for human immunodeficiency virus (HIV)-1 infection typically comprises a ‘backbone’ – two nucleoside analogue reverse transcriptase inhibitors (NRTI) – and a third drug – either a non-nucleoside reverse transcriptase inhibitor (NNRTI), a protease inhibitor (PI) or an integrase strand-transfer inhibitor (INSTI).

The United States Department of Health and Human Services (DHHS) guidelines form a major basis for HIV public policy in resource-rich settings. As of February 2013, an NRTI backbone of tenofovir-emtricitabine/lamivudine, with either efavirenz (NNRTI), raltegravir (INSTI), or ritonavir-boosted atazanavir or darunavir (PI) comprises ‘Preferred’ initial therapy [1]. When to commence cART remains guided by the clinical stage and CD4 lymphocyte count; pre-treatment plasma HIV viral load was removed as an indication for starting cART in 2007. While such recommendations arise from serial evaluation of individual studies by expert bodies, a systematic review of outcomes across multiple studies may reveal characteristics associated with success/failure, and so inform drug development, future study design, treatment guidelines and ultimately patient care.

Of the available meta-analyses of initial cART, most focus upon specific comparisons, or earlier studies [2], [3], [4], [5], [6]. None has evaluated outcomes beyond 48 weeks or by the regimen type (‘Preferred’ vs. ‘Alternative’). Much data (some of it unpublished) have been generated since the last broad-ranging analysis of initial cART efficacy [4], and an updated comprehensive assessment of initial cART efficacy and its associations is warranted.

## Methods

This systematic review evaluated all prospective studies of initial cART in adults reported through December 31, 2012. The primary outcome measure was antiviral efficacy, defined as undetectable (study-defined) plasma HIV viral load reported on an intention-to-treat (study-defined) basis. Substitution of any initial drug was regarded as treatment failure. Secondary outcomes were efficacy at weeks 48, 96 and 144, change in efficacy post-week 48, and premature cessation of initial cART. Subgroup analyses of efficacy were performed within pre-treatment HIV viral load strata (≥ or <100,000 copies/mL plasma) and by use of a ‘Preferred’ or ‘Alternative’ regimen as per the February 2013 edition of the DHHS guidelines. Additionally, we aimed to identify characteristics associated with heterogeneity of summarised efficacy, and premature treatment cessation due to participant decision, adverse events or virological failure.

### Study protocol and eligibility criteria

Conduct of this study was in accordance with the PRISMA Statement [7]. The protocol/analysis plan are available from the editors or authors upon request (**Protocol S1**).

This review aimed to include all studies of initial cART, subject to strict eligibility criteria to ensure data quality. Included studies: were conducted in consenting, treatment-naïve, HIV-1-infected adults; were a prospective cohort or randomised trial; reported efficacy data; and had a minimum of 48 weeks' follow-up. Comparative trials of initial cART were assessed only for the duration that the original randomisation was preserved.

We excluded studies of: retrospective or cross-sectional design; cART regimens categorised as not to be offered at any time in key treatment guidelines from 1996 through 2013 [1], [8], [9]; multiple/variable regimens within a single study arm; and directly-observed therapy. However, treatment groups with fixed, unspecified, dual-NRTI backbones and a common third drug were permitted [10]. Studies of novel, class-sparing regimens were considered for inclusion on an individual basis after review of the efficacy data (relative to standard triple-drug therapy based on a dual-NRTI backbone) by at least two authors. Boosting-dose ritonavir was not regarded an antiretroviral drug (but included in pill counts/dosing requirements). Apart from excluding any study presenting <48 weeks of efficacy data, eligibility criteria were the same as our previous systematic review of pre-2008 studies [4].

### Data sources and search strategy

The search period was January 1, 2008 to December 31, 2012. Electronic databases searched were: MEDLINE; Cochrane Central Register of Controlled Trials; United States National Institutes of Health clinical trials registry; and the International Standard Randomised Controlled Trial Numbers registry. For each of these, the search strategy was: ‘(“drug”) AND (HIV OR antiretroviral) AND (cohort OR randomised trial)’, where “drug” was the generic or pre-approval code name of an antiretroviral drug. No language restriction was applied. Abstracts from the following key scientific meetings between 2008 and 2012 were also searched: Conference on Retroviruses and Opportunistic Infections; International AIDS Society; Interscience Conference on Antimicrobial Agents and Chemotherapy; and the International Congress on Drug Therapy in HIV. Product labels and medical reviews of antiretroviral drugs published by the United States Food and Drug Administration and the European Medicines Agency between January 1, 1996 and December 31, 2012 were reviewed manually.

The above data were combined with all eligible 1995–2008 studies included in our earlier systematic review [4]. All were manually reviewed in duplicate by an author (FJL) for eligibility before being combined with results of the latest search. Discrepancies were discussed with a second author (AC).

Study synopses accessible on the following pharmaceutical company websites up to December 31, 2012 were reviewed: Abbott; Boehringer-Ingelheim; Bristol-Myers Squibb; Gilead Sciences; GlaxoSmithKline; and Roche. Other antiretroviral manufacturers did not have such websites.

All manufacturers were approached for relevant data missing from the above sources; data were provided by Bristol-Myers Squibb (August 15, 2012), Gilead Sciences (August 10, 2012), MSD (July 31, 2012), and ViiV Healthcare (November 29, 2012).

**Figure 1:**
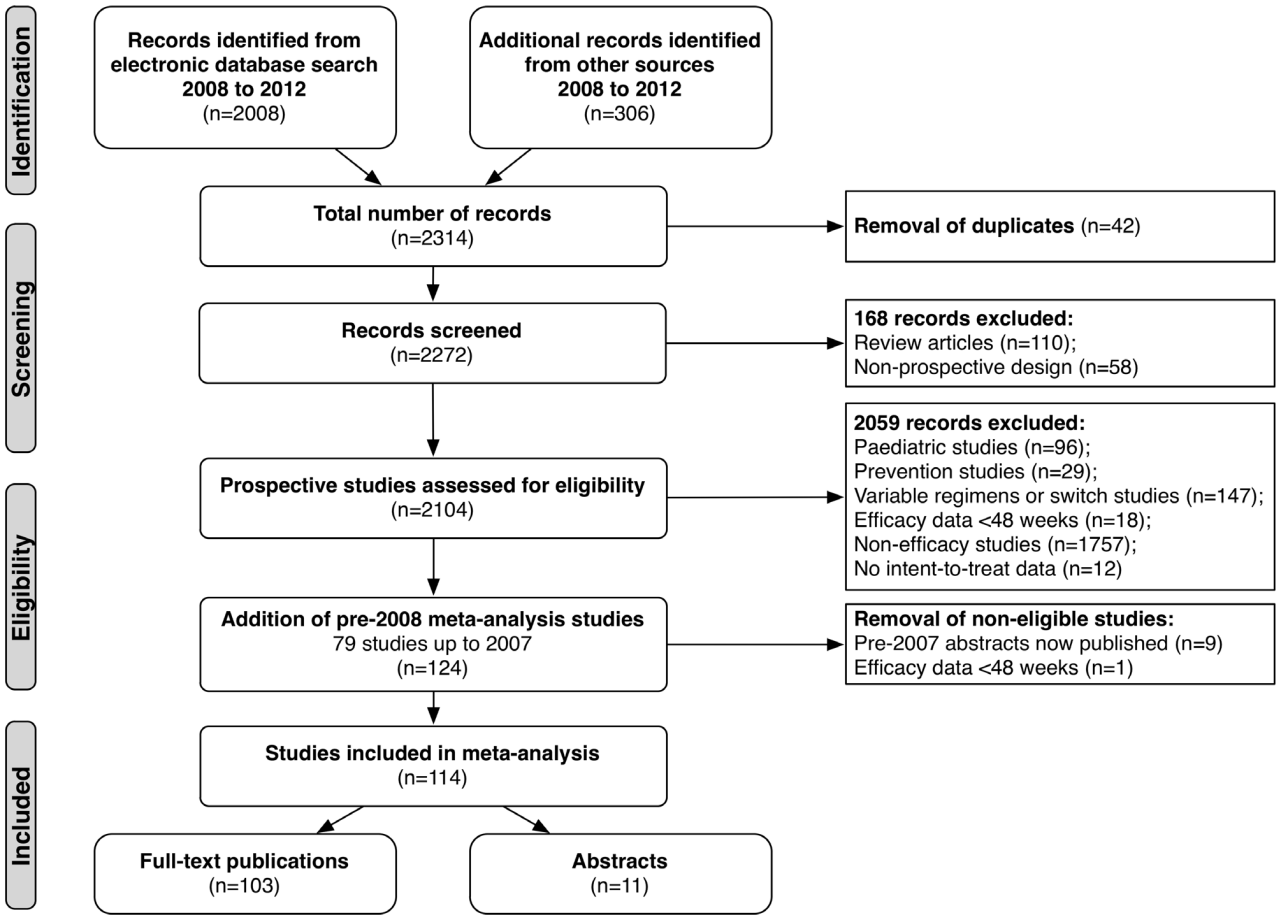
PRISMA statement 2009 flow chart. Diagram depicts each step of the study selection process undertaken in this systematic review and meta-analysis, including the reasons for exclusion.

The PRISMA flow diagram is depicted in **Figure 1**.

### Data extraction

Study arms were regarded as individual groups. For every group, characteristics collected were: year of commencement; participant numbers; latest reporting format; study characteristics; eligibility criteria; participant/disease characteristics; treatment characteristics; efficacy; and rates of premature treatment cessation. Efficacy data were collected cumulatively through study weeks 48, 96 and 144.

Dosing requirements were assumed to be in accordance with the product label at the time of study initiation or subsequent approval, unless otherwise stated. Efficacy data reported using the ‘time to loss of virological response’ (TLOVR) algorithm were collected preferentially. The newer ‘Snapshot’ algorithm was not considered, due to the small numbers and reported similarity of results to TLOVR [11]. All plasma viral load assays were considered equivalent – when more than one threshold was reported (e.g. both <400 and <50 copies/mL plasma), the lowest was used.

Data were abstracted manually in triplicate by one author (FJL) using a standardised data collection form. Ethnicity was recorded as ‘white’, ‘black’ or ‘other’, defined as previously [4]. Cause of premature treatment cessation was categorised as one of: adverse event, participant decision (withdrawal, loss to follow-up), virological failure, and other. No imputation was made for missing data (marked as unavailable). Completed forms and the resulting electronic database were audited by a second author (AC) to ensure data quality.

### Summary measures and data synthesis

The study arm was the unit of analysis, with clustering by trial accounted for. Principal summary measures were the proportion of participants per group and the mean (for continuous variables, including proportions). For summary measures across study arms, outcomes were expressed as a mean percentage, weighted for group size. Standard error of the mean was used to adjust for group size differences and heterogeneity was quantified using the *I* ^2^ statistic [12]. Adjusted absolute difference in outcomes within categorical variables was expressed as a percentage coefficient. Differences between means were compared using Student's *t*-test.

A multivariable, random-effects, linear regression approach was used to explore sources of heterogeneity in measures of efficacy and treatment cessation. Variables pre-selected for testing included all study, participant, disease and treatment characteristics as well as adverse events (**Table 1**). Year of study commencement was excluded from the primary predictor analysis, because of its likely relationship to other variables (drug potency/tolerability, pill/dose counts, availability of genotypic testing, emphasis on maximum pill adherence). Fixed NRTI backbones and third drug classes were compared, not individual drugs. This was done to retain analytical power and sensitivity across subgroup analyses. Furthermore, comparative prospective trials are powered for non-inferiority, and no clear superiority has hitherto been demonstrated for individual third drugs within a given class for initial cART – excepting boosted vs. unboosted PI. Unboosted PIs, (including unboosted atazanavir) were considered here as a separate class. Backwards, stepwise selection was used: only those statistically significant (*p*≤0.05) in univariable analysis were further assessed in multivariable models. Additionally, where covariate data were missing for >20% of individuals within a group, that group was excluded for assessment of that covariate.

**Table 1:** Characteristics included in linear regression analyses.

Study design	Participant pre-treatment characteristics	Treatment characteristics	Safety outcomes
study phase	risk factors for HIV infection	daily pill count	serious adverse events
placebo-controlled	sex	daily dose count	grade 2 to 4 clinical adverse events*
geography of recruitment	prior CDC category C events	dosing requirements relative to food	grade 3 to 4 laboratory adverse events*
intention-to-treat strategy	race	NRTI backbone	
study eligibility criteria (haemoglobin, liver function, CD4 count, HIV-1 plasma viral load, pre-treatment genotyping)	pre-treatment HIV-1 plasma viral load	third drug class	
	pre-treatment CD4 count		
	hepatitis B or C co-infection		

*Moderate or greater severity – graded according to 2009 Division of AIDS Classification.

CDC, Centers for Disease Control and Prevention; HIV, human immunodeficiency virus; NRTI, nucleoside reverse transcriptase inhibitor.

For stratified antiviral efficacy (pre-treatment viral load; regimen type) tests for interaction were conducted first. Only variables with statistically significant interactions with the stratified outcome were further interrogated using the aforementioned linear regression approach.

The following pre-planned sensitivity analyses were performed for the predictor linear regression models: (1) including only recent data by excluding studies commenced prior to 2005; (2) excluding cohorts and abstracts to correct for potential differences in study/report quality; and (3) including year of commencement as a variable. Four post-hoc analyses were performed: (1) descriptive efficacy/premature cessation data for groups using tenofovir-emtricitabine and abacavir-lamivudine, both overall and stratified by pre-treatment plasma viral load ≥ and <100,000 copies/mL; (2) a variant of the primary linear regression model for overall efficacy conducted after exclusion of all abacavir-lamivudine groups without HLA-B*5701 screening; (3) descriptive efficacy/premature cessation data and a linear regression model for overall efficacy when considering raltegravir separately from other INSTI drugs; and (4) descriptive efficacy/premature cessation data and a linear regression model for overall efficacy when considering unboosted atazanavir separately from other unboosted PIs (**Tables S1-S6 in Appendix S1: Supplementary post-hoc stratified analyses**).

All analyses were performed using STATA, Version 11 (StataCorp LP, College Station, Texas, USA). The *meta* suite of commands were used for combining data across trial arms.

## Results

### Study selection

The search (2008-2012) yielded 2,272 studies (2,008 publications, 306 abstracts), with 42 duplicates; 45 studies met the eligibility criteria. Following review and addition of pre-2008 studies (removal of duplicates and one pre-2008 cohort with only 24 weeks of follow-up), 114 studies (103 publications, 11 abstracts) were included (**Table 2**).

**Table 2:** Included studies and treatment groups.

Study title (Name) [Reference]	Study type	Placebo control	Included treatment groups [n = ]	Duration (weeks)
**1100.1486 (VERxVE)** [*Antivir Ther* 2011; 16: 759–69]	RCT	Yes	TDF-FTC+NVP 400 mg once-daily [505] vs. TDF-FTC+NVP 200 mg twice-daily [508]	48
**2NN** [*Lancet* 2004; 363: 1253–63.]	RCT	No	d4T-3TC+EFV [381] vs. d4T-3TC+NVP [378]	48
**A4001026 (MERIT)** [*J Infect Dis* 2010; 201: 803–13]	RCT	Yes	AZT-3TC+EFV [311] vs. AZT-3TC+MVC [303]	96
**A4001078** [*6th IAS Conference* 2011 (abstract TUAB0103)]	RCT	No	rAZV+MVC [60] vs. TDF-FTC+rAZV [61]	96
**A5271015** [*6th IAS Conference* 2011 (abstract TUAB0101)]	RCT	Yes	TDF-FTC+LRV [130] vs. TDF-FTC+EFV [63]	48
**ABCDE** [*J Acquir Immun Defic Syndr* 2007; 44: 139–47]	RCT	No	ABC-3TC+EFV [115] vs. d4T-3TC+EFV [122]	96
**ACH443-015** [*CROI* 2010 (abstract K-131)]	RCT	Yes	TDF-ELV+EFV [39] vs. TDF-3TC+EFV [37]	96
**ACTG 384** [*N Engl J Med* 2003; 349: 2293–303]	RCT	Yes	AZT-3TC+EFV [155] vs. AZT-3TC+NFV [155] vs. d4T-ddI+EFV [155] vs. d4T-ddI+NFV [155]	96
**ACTG 5095** [*N Engl J Med* 2004; 350: 1850–61]	RCT	Yes	AZT-3TC+EFV [382]	144
**ACTG A5073** [*Clin Infect Dis* 2010; 50: 1041–52]	RCT	No	2 NRTIs+rLPV twice-daily [159] vs. 2 NRTIs+rLPV once-daily [161]	48
**ACTG A5142** [*N Engl J Med* 2008; 358: 2095–106]	RCT	No	2 NRTIs+EFV [250] vs. 2 NRTIs+rLPV [253] vs. rLPV+EFV [250]	96
**ACTG A5175 (PEARLS)** [*PLoS Med* 2012; 9: e1001290]	RCT	No	TDF-FTC+EFV [526] vs. AZT-3TC+EFV [519]	96
**ACTG A5202** [*Ann Intern Med* 2011; 154: 445–56]	RCT	Yes	ABC-3TC+EFV [465] vs. ABC-3TC+rAZV [463] vs. TDF-FTC+EFV [464] vs. TDF-FTC+rAZV [465]	144
**ACTG A5208 (OCTANE 2)** [*PLoS Med* 2012; 9: e1001236]	RCT	No	TDF-FTC+NVP [249] vs. TDF-FTC+rLPV [251]	144
**ACTG A5262** [*AIDS* 2011; 25: 2113–22]	Cohort	No	rDRV+RAL [112]	48
**Advanced HIV Mexico** [*J Acquir Immun Defic Syndr* 2010; 53: 582–8]	RCT	No	AZT-3TC+EFV [95] vs. AZT-3TC+rLPV [94]	48
**Advanz** [*AIDS Res Hum Retroviruses* 2010; 26: 747–57]	RCT	No	AZT-3TC+EFV [34] vs. AZT-3TC+rIDV [31]	144
**AI424-007** [*J Acquir Immun Defic Syndr* 2003; 32: 18–29]	RCT	Yes	d4T-ddI+AZV [103] vs. d4T-ddI+NFV [103]	48
**AI424-008** [*AIDS* 2003; 17: 2603–14]	RCT	Yes	d4T-3TC+AZV [181] vs. d4T-3TC+NFV [91]	48
**AI424-034** [*J Acquir Immun Defic Syndr* 2004; 36: 1001–19]	RCT	Yes	AZT-3TC+AZV [404] vs. AZT-3TC+EFV [401]	48
**AI424-089** [*J Acquir Immun Defic Syndr* 2008; 47: 161–7]	RCT	No	d4T-3TC+rAZV [95] vs. d4T-3TC+AZV [104]	96
**AI424-138** (CASTLE) [*Lancet* 2008; 372: 646–55]	RCT	No	TDF-FTC+rAZV [438] vs. TDF-FTC+rLPV [440]	96
**AI454-148** [FDA Product Label (Videx): http://www.accessdata.fda.gov/drugsatfda_docs/label/2006/020154s50,20155s39,20156s40,21183s16lbl.pdf]	RCT	No	AZT-3TC+NFV [253] vs. d4T-ddI+NFV [503]	48
**AI454-152** [*J Acquir Immun Defic Syndr* 2002; 31: 399–403]	RCT	No	AZT-3TC+NFV [253] vs. d4T-ddI+NFV [258]	48
**AI455-096** [*43rd Annual ICAAC* 2003 (abstract H843)]	RCT	Yes	d4T-3TC once-daily+EFV [74] vs. d4T-3TC twice-daily+EFV [76]	96
**AI455-099** [*43rd Annual ICAAC* 2003 (abstract H843)]	RCT	Yes	d4T-3TC once-daily+EFV [392] vs. d4T-3TC twice-daily+EFV [391]	96
**Altair** [*Clin Infect Dis* 2010; 51: 855–64]	RCT	No	TDF-FTC+EFV [114] vs. TDF-FTC+rAZV [105] vs. TDF-FTC+ABC-3TC [103]	48
**ANRS 12-04 (IMEA 011)** [*AIDS* 2003; 17: 1017–22]	Cohort	No	ddI-3TC+EFV [40]	48
**ANRS 129 (BKVIR)** [*50th Annual ICAAC* 2010 (abstract H232)]	Cohort	No	TDF-FTC+EFV [69]	48
**APV109141** [*HIV Clin Trials* 2009; 10: 356–67]	RCT	No	ABC-3TC+rFPV once-daily [106] vs. ABC-3TC+rFPV twice-daily [106]	48
**APV30001 (NEAT)** [*J Acquir Immun Defic Syndr* 2004; 35: 22-32]	RCT	No	ABC-3TC+FPV [166] vs. ABC-3TC+NFV [83]	48
**APV30002 (SOLO)** [*AIDS* 2004; 18: 1529–37]	RCT	No	ABC-3TC+NFV [327] vs. ABC-3TC+rFPV [322]	48
**ARES** [*HIV Clin Trials* 2005; 5: 235–45]	RCT	No	d4T-ddI+NFV [26] vs. ddI-3TC+NVP [22] vs. ddI-3TC+rSQV [23]	48
**ARTEN** [*Antivir Ther* 2011; 16: 339–48]	RCT	No	TDF-FTC+NVP [376] vs. TDF-FTC+rAZV [193]	48
**AVANTI 2** [*AIDS* 2000; 14: 367–74]	RCT	Yes	AZT-3TC+IDV [52]	48
**BI1046 (INCAS)** [*JAMA* 1998; 279: 930–7]	RCT	Yes	AZT-ddI+NVP [51]	48
**BI1129 (ATLANTIC)** [*AIDS* 2003; 17: 987–99]	RCT	No	d4T-ddI+3TC [104] vs. d4T-ddI+IDV [94] vs. d4T-ddI+NVP [85]	96
**BI1182.33** [*8th International Congress on Drug Therapy* 2006 (abstract PL13.4)]	RCT	No	TDF-3TC+rLPV [185] vs. TDF-3TC+r(100 mg)TPV [187] vs. TDF-3TC+r(200 mg)TPV [186]	48
**BMS-001 (START 1)** [*AIDS* 2000; 14: 1591–600]	RCT	Yes	AZT-3TC+IDV [103] vs. d4T-3TC+IDV [101]	48
**BMS-002 (START 2)** [*AIDS* 2000; 14: 1601–10]	RCT	Yes	AZT-3TC+IDV [103] vs. d4T-ddI+IDV [102]	48
**CCTG589** [*6th IAS Conference* 2011 (abstract CDB336)]	RCT	No	rLPV+RAL [26] vs. TDF-FTC+EFV [25]	48
**Chelsea Westminster** [*Antivir Ther* 2006; 11: 73–8]	RCT	No	AZT-3TC+EFV [56]	48
**CNA109586 (ASSERT)** [*J Acquir Immun Defic Syndr* 2010; 55: 49–57]	RCT	No	ABC-3TC+EFV [192] vs. TDF-FTC+EFV [193]	96
**CNA30021 (ZODIAC)** [*J Acquir Immun Defic Syndr* 2005; 38: 417–25]	RCT	Yes	ABC-3TC once-daily+EFV [384] vs. ABC twice-daily-3TC+EFV [386]	48
**CNA30024** [*Clin Infect Dis* 2004; 39: 1038–46]	RCT	Yes	ABC-3TC+EFV [324] vs. AZT-3TC+EFV [325]	48
**CNAAB3005** [*JAMA* 2001; 285: 1155–63]	RCT	Yes	AZT-3TC+ABC [262] vs. AZT-3TC+IDV [264]	96
**CNAB3014** [*Curr Med Res Opin* 2004; 20: 1103–14]	RCT	No	AZT-3TC+ABC [169] vs. AZT-3TC+IDV [173]	48
**CNAF3007** [*Antivir Ther* 2003; 8: 163–71]	RCT	No	AZT-3TC+ABC [98] vs. AZT-3TC+NFV [97]	48
**COL100758** [*AIDS Res Hum Retroviruses* 2009; 25: 395–403]	RCT	No	ABC-3TC+r(100 mg)FPV [58] vs. ABC-3TC+r(200 mg)FPV [57]	96
**COL102060** (SHARE) [*HIV Clin Trials* 2008; 9: 152–63]	Cohort	No	ABC-3TC+rAZV [111]	48
**COL103952 (ALERT)** [Smith KY, et al. *AIDS Res Ther* 2008; 5: 5]	RCT	No	TDF-FTC+rAZV [53] vs. TDF-FTC+rFPV [53]	48
**COL111429 (SHIELD)** [*HIV Clin Trials* 2010; 11: 260–9]	Cohort	No	ABC-3TC+RAL [35]	96
**COMBINE** [*Antivir Ther* 2002; 7: 81–90]	RCT	No	AZT-3TC+NFV [70] vs. AZT-3TC+NVP [72]	48
**CTN 177** [*J Acquir Immun Defic Syndr* 2009; 50: 335–7]	RCT	No	AZT-3TC+NVP [26] vs. AZT-3TC+rLPV [25]	96
**Danish** [*AIDS* 2003; 17: 2045–52]	RCT	No	AZT-3TC+rSQV [60] vs. d4T-ddI+ABC [60]	48
**DART** [*AIDS* 2006; 20: 1391–9]	Cohort	No	AZT-3TC+TDF [300]	48
**DMP 266-006** [*N Engl J Med* 1999; 341: 1865–73]	RCT	No	AZT-3TC+EFV [422] vs. AZT-3TC+IDV [415] vs. EFV+IDV [429]	144
**EARTH 2** [*J Acquir Immun Defic Syndr* 2000; 25: 26–35]	RCT	No	d4T-3TC+IDV [32]	48
**EPV20001** [*Clin Infect Dis* 2004; 39: 411–8]	RCT	Yes	AZT-3TC once-daily+EFV [278] vs. AZT-3TC twice-daily+EFV [276]	48
**EPZ104057 (HEAT)** [*AIDS* 2009; 23: 1547–56]	RCT	Yes	ABC-3TC+rLPV [343] vs. TDF-FTC+rLPV [345]	96
**ESS100327 (ACTION)** [*AIDS Res Ther* 2009; 6: 3]	RCT	No	AZT-3TC+ABC [139] vs. AZT-3TC+AZV [140]	48
**ESS100732 (KLEAN)** [*Lancet* 2006; 368: 476–82]	RCT	No	ABC-3TC+rFPV [443] vs. ABC-3TC+rLPV [444]	48
**ESS30009** [*J Infect Dis* 2005; 192: 1921–30]	RCT	No	ABC-3TC+EFV [169]	48
**ESS40001** (CLASS) [*J Acquir Immun Defic Syndr* 2006; 43: 284–92]	RCT	No	ABC-3TC+d4T [98] vs. ABC-3TC+EFV [97] vs. ABC-3TC+rFPV [96]	96
**ESS40002** [*HIV Med* 2006; 7: 85–98]	RCT	No	AZT-3TC+ABC [85] vs. AZT-3TC+NFV [88] vs. d4T-3TC+NFV [81]	96
**FOCUS** [*J Internat AIDS Soc* 2006; 8: 36]	RCT	No	2 NRTIs+EFV [83] vs. 2 NRTIs+rSQV [82]	48
**FTC301A** [*JAMA* 2004; 292: 180–90]	RCT	Yes	d4T-ddI+EFV [285] vs. d4T-FTC+EFV [286]	48
**GEMINI** [*J Acquir Immun Defic Syndr* 2009; 50: 367–74]	RCT	No	TDF-FTC+rSQV [167] vs. TDF-FTC+rLPV [170]	48
**GESIDA 3093** [*Clin Infect Dis* 2008; 47: 1083–92]	RCT	No	ddI-3TC+EFV [186] vs. AZT-3TC+EFV [183]	48
**GS-01-934** [*J Acquir Immun Defic Syndr* 2006; 43: 535–40]	RCT	No	TDF-FTC+EFV [244] vs. AZT-3TC+EFV [243]	144
**GS-99-903** [*JAMA* 2004; 292: 191–201]	RCT	Yes	TDF-3TC+EFV [299] vs. d4T-3TC+EFV [301]	144
**GS-US-216-0105** [*AIDS* 2011; 25: 1881–6]	RCT	Yes	TDF-FTC+cobicistat-AZV [50] vs. TDF-FTC+rAZV [29]	48
**GS-US-236-0102** [*Lancet* 2012; 379: 2439–48]	RCT	Yes	TDF-FTC+cobicistat-EVG [348] vs. TDF-FTC+EFV [352]	48
**GS-US-236-0103** [*Lancet* 2012; 379: 2429–38]	RCT	Yes	TDF-FTC+cobicistat-EVG [353] vs. TDF-FTC+rAZV [355]	48
**GS-US-236-0104** [*AIDS* 2011; 25: F7–12]	RCT	Yes	TDF-FTC+cobicistat-EVG [48] vs. TDF-FTC+EFV [23]	48
**GS-US-264-0110** [*J Int AIDS Soc* 2012; 15(S4): 18221]	RCT	No	TDF-FTC+EFV [392] vs. TDF-FTC+RPV [394]	48
**HIV-NAT 003** [*J Acquir Immun Defic Syndr* 2001; 27: 116–23]	RCT	No	AZT-3TC+ddI [53]	48
**ING112276 (SPRING-1)** [*Lancet Infect Dis* 2012; 12: 111–8]	RCT	No	2 NRTIs+DTG [155] vs. 2 NRTIs+EFV [50]	96
**LAKE** [*Antiviral Res* 2010; 85: 403–8]	RCT	No	ABC-3TC+EFV [63] vs. ABC-3TC+rLPV [63]	48
**LORAN** [*Open AIDS J* 2011; 5: 44–50]	RCT	No	AZT-3TC+rLPV [35]	48
**LOREDA** [*6th IAS Conference* 2011 (abstract CDB354)]	Cohort	No	3TC+rLPV [39]	48
**M02-418** [*J Acquir Immun Defic Syndr* 2006; 43: 153–60]	RCT	No	TDF-FTC+rLPV once-daily [115] vs. TDF-FTC+rLPV twice-daily [75]	96
**M05-730** [*J Acquir Immun Defic Syndr* 2009; 50: 474–81]	RCT	No	TDF-FTC+rLPV once-daily [333] vs. TDF-FTC+rLPV twice-daily [331]	96
**M10-336 (PROGRESS)** [*HIV Clin Trials* 2011; 12: 255–67]	RCT	No	TDF-FTC+rLPV [105] vs. rLPV+RAL [101]	96
**M97-720** [*AIDS* 2001; 15: 1–9]	Cohort	No	d4T-3TC+rLPV [100]	96
**M98-863** [*N Engl J Med* 2002; 346: 2039–46]	RCT	Yes	d4T-3TC+NFV [327] vs. d4T-3TC+rLPV [326]	48
**M99-056** [*J Infect Dis* 2004; 189: 265–72]	RCT	No	d4T-3TC+rLPV once-daily [19] vs. d4T-3TC+rLPV twice-daily [19]	48
**MK0518-004** [*J Acquir Immun Defic Syndr* 2007; 46: 125–33]	RCT	Yes	TDF-3TC+RAL [160] vs. TDF-3TC+EFV [38]	144
**MK0518-021 (STARTMRK)** [*Lancet* 2009; 374: 796–806]	RCT	Yes	TDF-FTC+RAL [281] vs. TDF-FTC+EFV [282]	144
**MK0518-071 (QDMRK)** [*Lancet Infect Dis* 2011; 11: 907–15]	RCT	Yes	TDF-FTC+RAL once-daily [382] vs. TDF-FTC+RAL twice-daily [388]	48
**MONARK** [*AIDS* 2008; 22: 385–93]	RCT	No	AZT-3TC+rLPV [53]	48
**N2R** [*Clin Infect Dis* 2009; 48: 1752–9]	RCT	No	d4T-3TC+EFV [71] vs. d4T-3TC+NVP [71]	48
**NEWART** [*Int J Clin Pract* 2011; 65: 1240–9]	RCT	No	TDF-FTC+NVP [75] vs. TDF-FTC+rAZV [77]	48
**Nigerian ARV Program Cohort** [*J Acquir Immun Defic Syndr* 2005; 40: 65–9]	Cohort	No	d4T-3TC+NVP [50]	48
**NORA (DART substudy)** [*HIV Med* 2010; 11: 334–44]	RCT	Yes	AZT-3TC+ABC [300] vs. AZT-3TC+NVP [300]	48
**NVP China Cohort** [*PLoS One* 2008; 3: e3918]	Cohort	No	AZT-ddI+NVP [65] vs. d4T-3TC+NVP [69] vs. AZT-3TC+NVP [64]	48
**ONCE Cohort** [*Antivir Ther* 2002; 6: 249–53]	Cohort	No	ddI-3TC+EFV [75]	48
**ONCE RCT** [*Antivir Ther* 2003; 8: 339–46]	RCT	No	AZT-3TC+EFV [34] vs. AZT-3TC+NFV [34]	48
**OZCOMBO 1** [*AIDS* 2000; 14: 1171–80]	RCT	No	AZT-3TC+IDV [35] vs. d4T-3TC+IDV [34] vs. d4T-ddI+IDV [37]	48
**OZCOMBO 2** [*HIV Clin Trials* 2002; 3: 177–85]	RCT	No	AZT-3TC+NVP [20] vs. d4T-3TC+NVP [22] vs. d4T-ddI+NVP [23]	48
**PROAB3001** [*Antivir Ther* 2000; 5: 215–25]	RCT	Yes	AZT-3TC+APV [116]	48
**QUAD** [*J Antimicrob Chemother* 2005; 55: 246–51]	RCT	No	AZT-3TC+EFV [26]	48
**SCAN** [*AIDS* 2000; 14: 2485–94]	RCT	No	d4T-ddI+NVP once-daily [45] vs. d4T-ddI+NVP twice-daily [44]	48
**SENC** [*HIV Clin Trials* 2002; 3: 186–94]	RCT	No	d4T-ddI+EFV [31] vs. d4T-ddI+NVP [36]	48
**South African Workplace HIV Program** [*17th AIDS Conference* 2008 (abstract MOPE0046)]	Cohort	No	AZT-3TC+EFV [1416]	144
**Spanish ddI-3TC-NVP QD** [*Antivir Ther* 2005; 10: 605–14]	Cohort	No	ddI-3TC+NVP [70]	48
**Swiss Ritonavir** [*J Acquir Immun Defic Syndr* 2000; 23: 17–25]	RCT	No	AZT-3TC+r(1200 mg) [23]	96
**Thai Indinavir** [*Antivir Ther* 2005; 10: 911–6]	Cohort	No	d4T-3TC+rIDV [80]	96
**TMC114-C211 (ARTEMIS)** [*AIDS* 2008; 22: 1389–97]	RCT	No	TDF-FTC+rDRV [343] vs. TDF-FTC+rLPV [346]	96
**TMC125 VIR2038 (SENSE)** [*AIDS* 2011; 25: 2249–58]	RCT	Yes	2 NRTIs+ETR [79] vs. 2 NRTIs+EFV [78]	48
**TMC278-C204** [*AIDS* 2010; 24: 55–65]	RCT	No	2 NRTIs+RPV [279] vs. 2 NRTIs+EFV [89]	144
**TMC278-TiDP6-C209/215 (ECHO/THRIVE)** [*J Acquir Immun Defic Syndr* 2012; 60: 33–42]	RCT	Yes	2 NRTIs+RPV [686] vs. 2 NRITs+EFV [682]	96
**VACH** [*J Antimicrob Chemother* 2009; 63: 189–96]	Cohort	No	AZT-3TC+EFV [409] vs. ddI-3TC+EFV [219]	144
**VESD** [*HIV Clin Trials* 2005; 6; 320–8]	Cohort	No	ddI-3TC+EFV [167]	48
**VIRGO** [*Antivir Ther* 2000; 5: 267–72]	Cohort	No	d4T-ddI+NVP [100]	48

ABC, abacavir; AZT, zidovudine; APV, amprenavir; AZV, atazanavir; d4T, stavudine; ddI, didanosine; DRV, darunavir; DTG, dolutegravir; EFV, efavirenz; ELV, elvucitabine; ETR, etravirine; EVG, elvitegravir; FPV, fosamprenavir; FTC, emtricitabine; IDV, indinavir; LPV, lopinavir; MVC, maraviroc; NFV, nelfinavir; NVP, nevirapine; r, ritonavir; RPV, rilpivirine; RAL, raltegravir; SQV, saquinavir; TDF, tenofovir; TPV, tipranavir.

### Study and participant characteristics

Of 114 included studies, 97 (85%) were randomised trials and 17 (15%) prospective cohorts (**Table 3**), encompassing 216 treatment groups with 40,124 participants (median 112 participants/group; interquartile range 63 to 200). This represents 73 new groups (32 randomised trials, 3 cohorts, 17,057 participants) since our earlier review [4]. Participant and treatment characteristics are shown in **Table 4**; these were similar in terms of NRTI backbone and third drug class, demographics and disease stage for each analysis population (data not shown).

**Table 3:** Study characteristics.

Characteristics		Studies (n = 114)	Groups (n = 216)	Participants (n = 40,124)	Weighted proportion (%)
**Study design**					
**Randomised**	**no**	17	20	3,590	8.9
	**yes**	97	196	36,534	91.1
**Placebo**	**no**	79	148	22,494	56.1
	**yes**	35	68	17,630	43.9
**Phase** *	**2**	29	50	4,386	10.9
	**3**	48	96	26,325	65.6
	**4**	37	70	9,413	23.5
**Sponsorship**	**academic**	30	55	10,681	26.6
	**industry**	63	123	26,547	66.2
	**industry + academic**	21	38	2,896	7.2
**Year commenced**	**pre-1997**	6	10	641	1.6
	**1997**–**1999**	27	53	8,108	20.2
	**2000**–**2002**	23	43	9,331	23.3
	**2003**–**2005**	32	61	13,062	32.6
	**2006**–**2008**	16	31	6,597	16.4
	**2009**–	9	17	2,346	5.8
**Recruitment geography**	**Africa**	109	204	36,736	91.6
	**The Americas**	97	179	31,468	78.4
	**Asia**	104	194	35,100	87.5
	**Australia/Europe**	100	186	33,342	83.1
**Maximum follow-up**	**48 weeks**	73	131	20,165	50.3
	**96 weeks**	29	60	11,629	29.0
	**144 weeks**	12	25	8,330	20.8
**Viral load endpoint**	**<50**	103	196	34,500	86.0
	**other** †	11	20	5,624	14.0
**ITT analysis**	**ITT M = F**	36	62	7,127	17.8
	**ITT NC = F**	44	84	13,676	34.1
	**TLOVR**	34	70	19,321	48.2
**Publication type**	**abstract only**	11	22	5,257	13.1
	**journal**	103	194	34,867	86.9
**Eligibility criteria**					
**ALT/AST**	**restricted**	88	173	31,536	78.6
	**unrestricted**	26	43	8,588	21.4
**CD4 count**	**restricted**	64	122	19,428	48.4
	**unrestricted**	50	94	20,696	51.6
**Genotype**	**restricted**	31	62	12,563	31.3
	**unrestricted**	83	154	27,561	68.7
**Haemoglobin**	**restricted**	67	133	24,224	60.4
	**unrestricted**	47	83	15,900	39.6
**Viral load**	**restricted**	92	184	33,915	84.5
	**unrestricted**	22	32	6,209	15.5
**History of IDU**	**restricted**	2	5	223	0.6
	**unrestricted**	112	211	39,901	99.4
**Previous AIDS**	**restricted**	6	11	1,436	3.6
	**unrestricted**	108	205	38,688	96.4

*Prospective cohort studies were considered phase 4 studies.

† Includes <400, <200, <40 and <20 copies/mL.

ITT, intention-to-treat; M = F, missing equals failure; NC = F, non-completer equals failure; TLOVR, time to loss of virological response; ALT, alanine transaminase; AST, aspartate transaminase; IDU, injection drug use; AIDS, acquired immune deficiency syndrome.

**Table 4:** Treatment and participant characteristics: all groups.

Characteristics		Groups (n = 216)	Participants (n = 40,124)	Mean (across groups)	Mean (weighted)
**Antiretroviral therapy**					
**Pills per day, mean (SD)**		216	40,124	6.8 (3.8)	6.3 (3.6)
**Doses per day, mean (SD)**		216	40,124	2.0 (0.7)	2.0 (0.7)
**Dosing with food, %**	**fasting only**	59	9,754	27.3	24.3
	**fasting + food**	22	5,108	10.2	12.7
	**food only**	65	11,947	30.0	29.8
	**no relation to food**	70	13,315	32.4	33.2
**NRTI backbone, %**	**TDF-FTC**	47	11,001	21.7	27.4
	**AZT-3TC**	56	10,832	25.9	27.0
	**d4T-3TC**	27	3,988	12.5	9.9
	**ABC-3TC**	26	5,516	12.0	13.7
	**d4T-ddI**	20	2,349	9.3	5.9
	**Nil/2 NRTIs**	20	4,064	9.3	10.1
	**TDF-3TC**	7	1,092	3.2	2.7
	**ddI-3TC**	8	802	3.7	2.0
	**AZT-ddI**	2	116	0.9	0.3
	**ddI-FTC**	1	286	0.5	0.7
	**3TC**	1	39	0.5	0.1
	**TDF-ELV** *	1	39	0.5	0.1
**Third drug class, %**	**NNRTI**	94	19,512	43.5	48.6
	**PI (boosted)**	56	9,724	25.9	24.2
	**PI (unboosted)**	38	5,686	17.6	14.2
	**NRTI/2 NRTIs**	12	1,771	5.6	4.4
	**INSTI**	9	2,150	4.2	5.4
	**INSTI+PI**	3	239	1.4	0.6
	**NNRTI+PI**	2	679	0.9	1.7
	**CCR5/CCR5+PI**	2	363	0.9	0.9
**Participants at baseline**					
**Age, years (SD)**		207	39,524	37 (2.1)	37 (2.1)
**Gender, % (SD)**	**male**	213	40,022	76 (14)	76 (15)
**Race, % (SD)**	**white**	170	33,524	64 (22)	65 (17)
	**black**	166	33,803	25 (19)	27 (17)
	**other**	163	32,689	12 (21)	9.8 (14)
**HIV risk factor, % (SD)**	**MSM**	88	14,596	48 (20)	52 (19)
	**IDU**	104	18,287	12 (11)	9.8 (9.2)
	**heterosexual**	83	14,261	39 (18)	38 (15)
	**other**	80	13,939	5.2 (8.3)	4.7 (6.5)
**Previous AIDS event, % (SD)**		161	30,035	14 (19)	12 (13)
**Pre-treatment CD4, % (SD)**		214	40,048	265 (103)	248 (81)
**Pre-treatment HIV viral load**	**log**	210	39,808	4.8 (0.3)	4.9 (0.2)
	**% ≥100,000 cp/mL**	150	31,409	41 (14)	43 (11)
**Hepatitis B sAg +, % (SD)**		129	26,739	3.2 (2.8)	3.4 (2.3)
**Hepatitis C Ab +, % (SD)**		124	25,487	11 (11)	10 (8.8)
**Weight, kg (SD)**		112	22,196	71 (5.6)	71 (5.1)

*ELV has not received regulatory approval. This group was excluded from the analysis.

SD, standard deviation; TDF, tenofovir; FTC, emtricitabine; AZT, zidovudine; 3TC, lamivudine; d4T, stavudine; ddI, didanosine; ABC, abacavir; ELV, elvucitabine; NRTI, nucleoside reverse transcriptase inhibitor; NNRTI, non-nucleoside reverse transcriptase inhibitor; PI, protease inhibitor; INSTI, integrase strand transfer inhibitor; CCR5, chemokine receptor 5 inhibitor; HIV, human immunodeficiency virus; MSM, men who have sex with men; IDU, injecting drug use; AIDS, acquired immune deficiency syndrome; Ab, antibody; sAg, surface antigen; cp, copies.

### Overall efficacy: all studies

**Figure 2:**
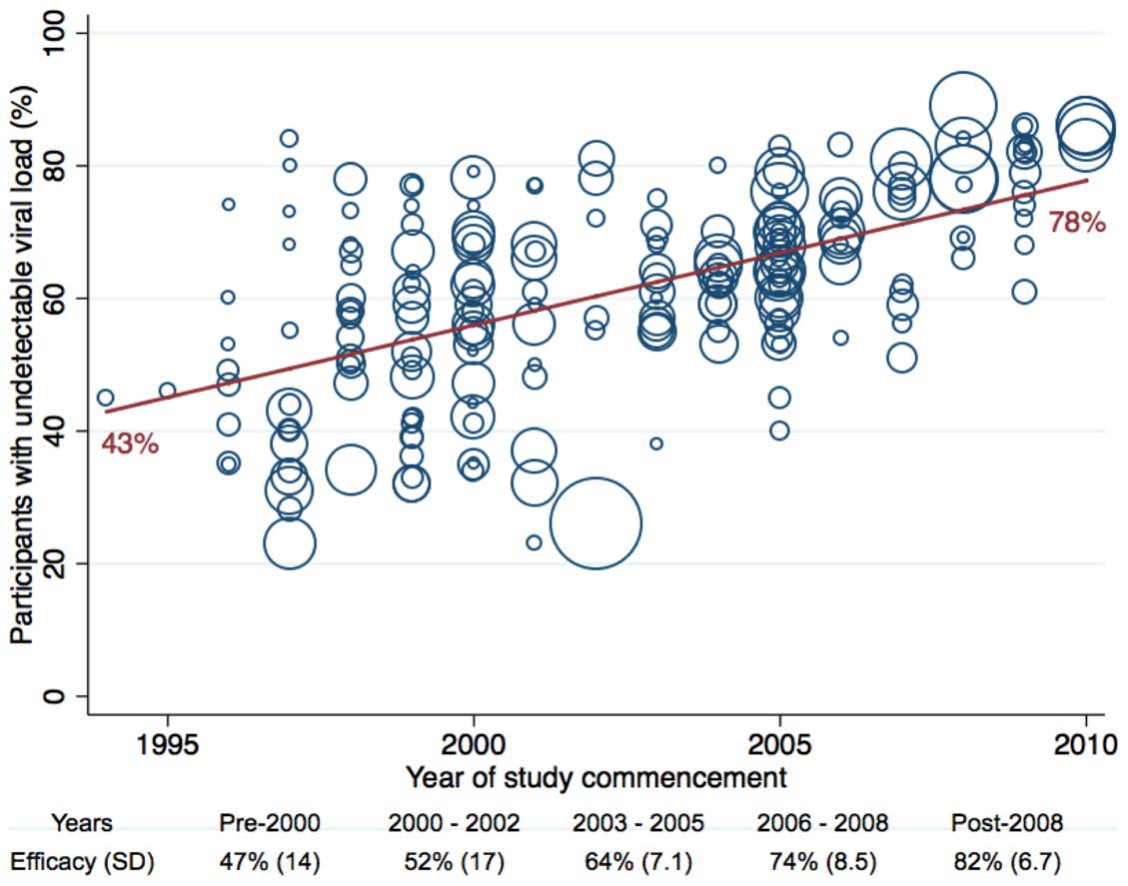
Efficacy by year of study commencement. Summary bubble plot displaying the change in weighted intention-to-treat antiviral efficacy of initial antiretroviral therapy by the year included studies were commenced. Each bubble represents an individual treatment group, proportional to size. SD, standard deviation.

Mean overall efficacy was 60% (SD 16) after a mean follow-up of 82 weeks (SD 38) with greater efficacy in more recent studies (**Table 5** **,** **Figure 2**). Collected data were highly heterogeneous (*I* ^2^ = 96%).

**Table 5:** Efficacy and categories of premature treatment cessation.

	All groups	Maximum duration of follow-up			Pre-treatment HIV viral load (copies/mL plasma)		DHHS recommendation (February 2013)		
		48 weeks	96 weeks	144 weeks	<100,000	≥100,000	Preferred*	Alternative*	Non-Preferred/Non-Alternative*
**Groups (participants)**	216 (40,124)	216 (40,124)	85 (19,959)	25 (8,330)	98 (13,184)	98 (9,694)	27 (5,677)	36 (8,556)	153 (25,891)
**Mean follow-up, weeks (SD)**	82 (38)	48 (-)	96 (-)	144 (-)	81 (36)	81 (36)	99 (41)	86 (35)	77 (37)
**Antiviral efficacy, % (SD)**	60 (16)	66 (16)	60 (16)	52 (18)	70 (15)	62 (15)	75 (7.9)	65 (6.6)	55 (17)
**Premature treatment cessation, % (SD)** †									
**Participant decision**	11 (6.6)	8.5 (4.8)	14 (7.9)	15 (8.5)	No data	No data	9.0 (4.6)	11 (5.2)	11 (7.3)
**Adverse events**	8.1 (5.9)	6.9 (5.4)	7.5 (5.3)	10 (5.6)	No data	No data	5.3 (2.8)	7.3 (4.0)	9.0 (6.7)
**Virological failure**	3.5 (4.0)	2.4 (3.0)	4.8 (4.9)	4.4 (2.7)	No data	No data	3.6 (4.4)	2.8 (3.5)	3.7 (4.0)
**Other**	4.7 (4.1)	3.3 (3.2)	6.4 (4.2)	8.3 (6.2)	No data	No data	3.4 (2.7)	4.5 (2.2)	5.0 (4.8)
**Total**	25 (11)	20 (9.0)	28 (11)	34 (9.6)	No data	No data	20 (8.1)	25 (7.8)	27 (12)

*According to United States Department of Health and Human Services February 2013 Adult and Adolescent Guidelines.

† Data for premature cessation stratified by pre-treatment viral load not available.

SD, standard deviation.

Study phase, intention-to-treat analysis method, genotype/CD4 eligibility restrictions, NRTI backbone, third drug class, daily pill and dose counts were identified as the primary sources of efficacy heterogeneity on univariable analysis (**Table 6**). In the multivariable analysis, higher efficacy was associated with: NRTI backbone (favouring tenofovir-emtricitabine, *p*<0.001); third drug class (favouring INSTI, *p*<0.001); and studies using intention-to-treat algorithm (favouring ‘missing equals failure’, *p*<0.001). This model accounted for 56% of the variance (*r^2^*) in reported efficacy.

**Table 6:** Characteristics* associated with efficacy of initial antiretroviral therapy: all groups.

			Univariable Analysis					Multivariable Analysis†			
		Efficacy, % (SD)	Coefficient, %‡	95%CI	*p*	*p* trend	*r* ^2^ (%)	Coefficient, %‡	95%CI	*p*	*p* group
**Study design**											
**Phase**	**2**	64 (15)	Ref								
	**3**	62 (16)	−6.0	−11.0, −0.9	0.022						
	**4**	54 (16)	−9.0	−14.5, −3.5	0.001	0.006	3.8				
**ITT analysis**	**ITT M = F**	53 (17)	Ref					Ref			
	**ITT NC = F**	59 (15)	−4.7	−9.7, 0.3	0.064			−6.4	−10.1, −2.7	0.001	
	**TLOVR**	64 (15)	1.1	−4.0, 6.2	0.662	0.035	2.1	−7.5	−11.5, −3.6	<0.001	<0.001
**Eligibility criteria**											
**Genotype**	**restricted**	72 (10)	Ref								
	**unrestricted**	55 (15)	−14.5	−18.5, −10.6	<0.001	<0.001	21.7				
**CD4 count**	**restricted**	54 (18)	Ref								
	**unrestricted**	66 (11)	6.9	3.0, 10.8	0.001	0.001	6.2				
**Treatment**											
**Pills per day**			−1.7	−2.2, −1.2	<0.001	<0.001	19.2				
**Doses per day**			−9.0	−11.5, −6.6	<0.001	<0.001	22.3				
**NRTI backbone**	**TDF-FTC**	73 (10)	Ref					Ref			
	**AZT-3TC**	48 (15)	−19.6	−24.4, −14.7	<0.001			−13.3	−18.0, −8.5	<0.001	
	**d4T-3TC**	55 (12)	−15.0	−21.0, −9.1	<0.001			−11.4	−16.8, −5.9	<0.001	
	**ABC-3TC**	63 (6.8)	−10.4	−16.3, −4.5	0.001			−7.6	−12.7, −2.6	0.003	
	**d4T-ddI**	44 (13)	−25.3	−32.0, −18.7	<0.001			−18.4	−24.9, −12.0	<0.001	
	**Nil/2 NRTIs**	66 (15)	−6.1	−12.6, 0.3	0.063			−0.7	−6.9, 5.4	0.818	
	**TDF-3TC**	69 (4.6)	−2.6	−11.3, 6.2	0.567			−1.2	−8.5, 6.2	0.758	
	**ddI-3TC**	65 (8.4)	−8.3	−18.0, 1.5	0.097			−7.9	−16.6, 0.8	0.075	
	**AZT-ddI**	42 (3.5)	−30.5	−49.2, −11.7	0.002			−35.3	−51.9, −18.7	<0.001	
	**ddI-FTC**	78 (-)	5.1	−18.3, 28.5	0.668			7.9	−11.6, 27.4	0.426	
	**3TC**	67 (-)	−5.9	−33.2, 21.5	0.671	<0.001	35.3	−9.8	−33.9, 14.3	0.424	<0.001
**Third drug class**	**NNRTI**	61 (15)	Ref					Ref			
	**PI (boosted)**	67 (8.6)	2.9	−1.0, 6.8	0.140			−0.9	−4.7, 3.0	0.660	
	**PI (unboosted)**	42 (11)	−19.2	−23.7, −14.8	<0.001			−15.0	−19.4, −10.6	<0.001	
	**NRTI/2 NRTIs**	51 (12)	−13.3	−20.4, −6.3	<0.001			−10.7	−17.4, −4.1	0.002	
	**INSTI**	84 (4.7)	18.5	10.7, 26.2	<0.001			11.9	4.6, 19.2	0.002	
	**INSTI+PI**	64 (3.6)	0.9	−13.3, 15.2	0.897			−6.8	−20.7, 7.0	0.331	
	**NNRTI+PI**	40 (16)	−21.2	−36.8, −5.6	0.008			−27.3	−41.9, −12.6	<0.001	
	**CCR5/CCR5+PI**	61 (4.7)	−0.9	−17.4, 15.5	0.911	<0.001	43.0	−2.7	−17.5, 12.2	0.723	<0.001

*Study phase, genotype/CD4 eligibility restrictions, pills and doses per day were significant on univariable analysis but not multivariable. Co-infection with hepatitis B or C were excluded from the multivariable analysis because >20% of groups were missing data.

† Study sponsorship, placebo use, country/region of recruitment, haemoglobin/viral load/liver function eligibility restrictions, race, risk factors for HIV infection, sex, previous AIDS events, pre-treatment viral load/CD4 count, dosing relative to food, serious adverse events, and clinical/laboratory adverse events of at least moderate severity were variables not significant on univariable analysis.

‡ Coefficient represents the adjusted percentage difference in outcomes relative to a unit increase in any variable (or relative to the reference variable within a category).

SD, standard deviation; CI, confidence interval; ITT, intention-to-treat; M = F, missing equals failure; NC = F, non-completer equals failure; TLOVR, time to loss of virological response; TDF, tenofovir; FTC, emtricitabine; AZT, zidovudine; 3TC, lamivudine; d4T, stavudine; ddI, didanosine; ABC, abacavir; ELV, elvucitabine; NRTI, nucleoside reverse transcriptase inhibitor; NNRTI, non-nucleoside reverse transcriptase inhibitor; PI, protease inhibitor; INSTI, integrase strand transfer inhibitor; CCR5, chemokine receptor 5 inhibitor.

Tenofovir-emtricitabine was associated with higher efficacy than abacavir-lamivudine (coefficient 7.6%; 95% confidence interval [CI] 2.6 to 12.7; *p* = 0.003). Only two groups, both 96 weeks in duration, used pre-treatment HLA-B*5701 screening before administering abacavir; overall weighted efficacy of these abacavir groups was 55% (SD 13), vs. 63% (SD 6.4) for the 24 groups without HLA-B*5701 screening. Amongst third drug classes, INSTI use was associated with higher efficacy compared to either an NNRTI (coefficient 11.9%; 95%CI 4.6 to 19.2; *p* = 0.002) or a boosted PI (coefficient 12.7%; 95%CI 4.6 to 19.2; *p* = 0.001). There was no difference in efficacy of NNRTI compared to boosted PI regimens.

### Change in efficacy by follow-up duration

Efficacy at 96 and 144 weeks was reported for 85 (39%, 19,959 participants) and 25 (12%, 8,330 participants) groups, respectively. For these groups, mean efficacy at 48 weeks was 66% (SD 16), declining to 60% (SD 16) at week 96, then 52% (SD 18) at week 144; the mean fall in efficacy was 8.3% (SD 3.9) between weeks 48 and 96, with a further 6.3% (SD 5.3) decline between weeks 96 and 144.

Type of NRTI backbone (favouring tenofovir-emtricitabine, *p*<0.001), third drug class (favouring INSTI, *p*<0.001) and efficacy analysis method (favouring ‘missing equals failure’, *p* = 0.001), eligibility by pre-treatment resistance genotype (favouring restriction, *p* = 0.007) and CD4 lymphocyte count (favouring no restriction, *p* = 0.001) were each associated on multivariable analysis with greater efficacy at week 48. At week 96, greater efficacy was associated with: phase 2 studies (vs. phase 3 or 4, *p*<0.001) and no eligibility restriction for CD4 count (*p*<0.001). The *r^2^* values were 66% and 39% for weeks 48 and 96, respectively. Due to insufficient data, a stable multivariable model could not be generated for efficacy through week 144.

For those studies reporting efficacy data through to week 96, a multivariable analysis for the decline in efficacy between weeks 48 and 96 was performed. Lesser decline was associated with phase 2 studies (*p* = 0.002), placebo use (*p*<0.001), and NRTI backbone (*p* = 0.002), but there was no significant difference between use of tenofovir-emtricitabine and abacavir-lamivudine.

### Efficacy stratified by regimen type: February 2013 DHHS guidelines

A total of 63 groups (29%, 14,233 participants) were allocated either a 2013 DHHS ‘Preferred’ regimen (27 groups, 13%, 5,677 participants) or an ‘Alternative’ regimen (39 groups, 18%, 9,305 participants). Amongst the ‘Preferred’ regimens, 14 groups (2,729 participants) initiated efavirenz: efficacy 72% (SD 7.6) over 108 weeks (SD 40); nine groups (1,776 participants) initiated atazanavir/ritonavir: efficacy 75% (SD 7.3) over 87 weeks (SD 42); one group (343 participants) initiated darunavir/ritonavir: efficacy 79% over 96 weeks; and three groups (829 participants) initiated raltegravir: efficacy 82% (SD 7.9) over 99 weeks (SD 59).

‘Preferred’ regimen efficacy was 75% (SD 7.9) over a mean follow up of 99 weeks (SD 41), compared to 66% (SD 6.6) over 82 weeks (SD 35) with ‘Alternative’ regimens (difference 10%; 95%CI 7.6 to 15.4; *p*<0.001). This difference persisted when stratified by high (difference 10%; 95%CI 3.0 to 17.0; *p* = 0.006) and low (difference 16%; 95%CI 7.9 to 23.7; *p*<0.001) pre-treatment viral load strata.

The initial regimen type selected was found to significantly interact with the relationship of third drug class (*p* = 0.040), dosing requirements (*p* = 0.024), previous AIDS events (*p* = 0.003), and pre-treatment CD4 lymphocyte count (*p* = 0.049) on efficacy. On regression analysis (**Table 7**), the pre-treatment CD4 count was associated with efficacy within ‘Preferred’ regimens (coefficient <0.1%; 95%CI 0.0 to 0.1; *p* = 0.035); that is, efficacy was <0.1% greater for every one cell increase in the absolute CD4 count.

**Table 7:** Characteristics* associated with efficacy of initial antiretroviral therapy: February 2013 DHHS ‘Preferred’ regimens (27 groups).

				Univariable Analysis					Multivariable Analysis†			
		Groups (participants)	Efficacy, % (SD)	Coefficient, %	95%CI	*p*	*p* trend	*r* ^2^ (%)	Coefficient, %	95%CI	*p*	*p* group
**Dosing requirements**	**None**	3 (886)	71 (7.9)	Ref								
	**Fasting only**	9 (1,620)	71 (7.0)	3.1	−7.6, 13.8	0.552						
	**Food only**	10 (2,164)	77 (8.7)	7.6	−2.9, 18.1	0.147						
	**Fasting + food**	5 (1,007)	79 (4.5)	8.6	−3.4, 20.6	0.152	0.323	5.4				
**Third drug class**	**NNRTI**	14 (2,729)	72 (7.6)	Ref								
	**PI (boosted)**	10 (2,119)	75 (6.8)	3.2	−3.7, 10.2	0.351						
	**INSTI**	3 (829)	82 (7.9)	7.6	−2.5, 17.6	0.134	0.282	5.9				
**Participant**	**Previous AIDS**			0.0	−0.2, 0.2	0.784	0.784	−5.8				
	**Pre-treatment CD4**			<0.1	0.0, 0.1	0.035	0.035	17.6	<0.1	0.0, 0.1	0.035	0.035

*Dosing relative to food, third drug class, previous AIDS events and pre-treatment CD4 interacted significantly with DHHS regimen type to affect efficacy.

† Dosing relative to food, NRTI backbone, third drug class and previous AIDS events were not significantly associated with efficacy on univariable analysis.

DHHS, United States Department of Health and Human Services; SD, standard deviation; CI, confidence interval; TDF, tenofovir; FTC, emtricitabine; 3TC, lamivudine; NRTI, nucleoside reverse transcriptase inhibitor; NNRTI, non-nucleoside reverse transcriptase inhibitor; PI, protease inhibitor; INSTI, integrase strand transfer inhibitor.

Amongst ‘Alternative’ regimens (**Table 8**), previous AIDS events were associated with lesser efficacy (coefficient −0.4%; 95%CI −0.6 to −0.1; *p* = 0.011), INSTI use with greater efficacy (vs. NNRTI; coefficient 15.3%; 95%CI 8.7 to 21.9; *p*<0.001), and a requirement for fasting when taking medications was associated with lesser efficacy (vs. no dosing requirement; coefficient −10.6%; 95%CI −17.7 to −3.4; *p* = 0.005).

**Table 8:** Characteristics* associated with efficacy of initial antiretroviral therapy: February 2013 DHHS ‘Alternative’ regimens (39 groups).

				Univariable Analysis					Multivariable Analysis†			
		Groups (participants)	Efficacy, % (SD)	Coefficient, %	95%CI	*p*	*p* trend	*r* ^2^ (%)	Coefficient, %	95%CI	*p*	*p* group
**Dosing requirements**	**None**	15 (2,900)	68 (4.2)	Ref					Ref			
	**Fasting only**	3 (720)	57 (4.8)	−12.5	−21.7, −3.4	0.008			−10.6	−17.7, −3.4	0.005	
	**Food only**	18 (4,603)	64 (7.8)	−5.0	−10.0, 0.0	0.051			−4.4	−9.0, 0.2	0.060	
	**Fasting + food**	3 (1,082)	81 (4.6)	13.5	4.9, 22.1	0.003	<0.001	45.7	4.1	−2.7, 10.8	0.226	0.004
**Third drug class**	**NNRTI**	11 (3,160)	67 (7.8)	Ref					Ref			
	**PI (boosted)**	24 (5,361)	64 (5.6)	−1.5	−6.4, 3.3	0.527			0.3	−4.8, 5.3	0.919	
	**INSTI**	4 (784)	85 (2.3)	18.4	10.6, 26.4	<0.001	<0.001	51.7	15.3	8.7, 21.9	<0.001	<0.001
**Participant**	**Previous AIDS**			−0.7	−1.2, −0.3	0.003	0.003	25.9	−0.4	−0.7, −0.1	0.019	0.019
	**Pre-treatment CD4**			0.1	0.1, 0.1	<0.001	<0.001	76.5				

*Dosing relative to food, third drug class, previous AIDS events and pre-treatment CD4 interacted significantly with DHHS regimen type to affect efficacy.

† NRTI backbone and pre-treatment CD4 count were not significantly associated with efficacy on univariable analysis.

DHHS, United States Department of Health and Human Services; SD, standard deviation; CI, confidence interval; TDF, tenofovir; FTC, emtricitabine; 3TC, lamivudine; ABC, abacavir; NRTI, nucleoside reverse transcriptase inhibitor; NNRTI, non-nucleoside reverse transcriptase inhibitor; PI, protease inhibitor; INSTI, integrase strand transfer inhibitor.

### Efficacy stratified by pre-treatment viral load

For high (≥100,000 copies/mL) and low (<100,000 copies/mL) viral load strata, 98 groups (45%, 22,878 participants, mean 81 weeks' follow-up [SD 36]) had efficacy data available. Mean efficacy was 62% (SD 15) and 70% (SD 15), respectively (difference 8.4%; 95%CI 6.0 to 10.9; *p*<0.001).

Stratification by pre-treatment viral load showed no significant interaction with the relationship of any pre-selected variable upon efficacy (*p*>0.05), and univariable-multivariable analysis was not pursued. Despite this lack of interaction, ‘Preferred’ regimens (February 2013 DHHS guidelines) maintained greater efficacy at both higher and lower viral load strata; efficacy within these strata were 73% (SD 12) and 82% (SD 12), respectively (difference 9.1%; 95%CI 4.3 to 14.0; *p* = 0.001).

### Premature treatment cessation: all studies

**Figure 3:**
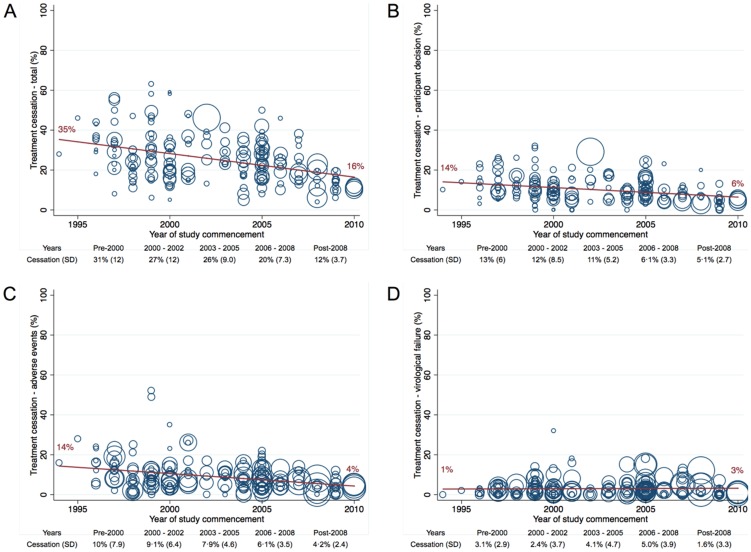
Premature cessation of initial antiretroviral therapy by year of study commencement. (A) Total cessation from all causes; (B) attributed to participant decision – withdrawal from study or lost to follow-up; (C) attributed to adverse events; and (D) due to study-defined virological failure. Studies were included in this analysis if total premature cessation was reported. SD, standard deviation.

An average of 25% (SD 11) of participants prematurely ceased initial cART. The most common reason was participant decision (11%, SD 6.6) followed by adverse events (8.1%, SD 5.9); virological failure was less common (3.5%, SD 4.0). Total premature cessation was lower with more recent studies (**Figure 3**). Most cessations (20%, SD 9.0) occurring through week 48, 9.2% (SD 11.0) ceasing between weeks 48 and 96, and a further 4.9% (SD 2.7) ceasing between weeks 96 and 144.

On multivariable analysis adjusting for study, participant, disease and treatment characteristics and adverse events, industry-only sponsorship was associated with lower cessation rates due to participant decision, compared to industry-supported academic (coefficient −2.6%; 95%CI −4.8 to −0.5; *p* = 0.015) and academic-only sponsorships (coefficient −4.0%; 95%CI −6.3 to −1.6; *p* = 0.001). Similar associations existed between sponsorship and cessation due to adverse events. Phase 2 studies were associated with lower rates of cessation due to adverse events than phase 3 or 4 studies (coefficients −2.6% and −4.0%, respectively; *p*<0.015). Neither NRTI backbone nor third drug class were associated with cessation attributed either to adverse events or participant decision.

### Studies commenced 2005 and later

The pre-planned sensitivity analysis of the most recent studies (commenced 2005 and later) included 82 groups. Of these, 76 (93%) were randomised trials and 6 (7%) were prospective cohorts, encompassing 16,795 participants (median 118 participants/group; interquartile range 47 to 170). Participant characteristics were similar to that of the primary analysis (mean age 37 years [SD 1.9]; mean CD4 count 238 cells/µL [SD 80]; mean HIV-1 plasma viral load log_10_ 4.9 [0.3]).

Mean overall efficacy was higher than for all included studies: 71% (SD9.6) over a mean follow-up of 86 weeks (SD 37). Efficacy at 96 and 144 weeks was reported for 37 (45%, 9,961 participants) and 12 (15%, 3,486 participants) groups, respectively. Mean efficacy at 48 weeks was 77% (SD 8.6), 70% (SD 11) at 96 weeks, and 66% (SD 6.3) at week 144. Total mean premature treatment cessation was 21% (SD 8.9). Like the primary analysis, participant decision (8.8%, SD 5.4) was the most common reason, followed by adverse events (6.5%, SD 4.0) and virological failure (4.3%, SD 4.3).

Multivariable analysis of studies commenced 2005 or later resulted in similar findings to the primary analysis. Tenofovir-emtricitabine continued to be associated with higher efficacy than abacavir-lamivudine (coefficient 8.8%; 95%CI 3.9 to 13.8; *p* = 0.001), as was INSTI over NNRTI (coefficient 5.5%; 95%CI 0.1 to 11.0; *p* = 0.050).

### Other sensitivity analyses

The separate analyses conducted after excluding cohorts and abstracts, and then including the year of commencement as a variable both gave similar results to the primary analysis (data not shown).

## Discussion

For studies initiated from 1995 through to 2010, the overall mean efficacy of initial cART was low (60% over 82 weeks), although it has risen substantially for more recent studies, suggesting it could rise further with studies commenced post-2010 (not available for this analysis before locking of the final database). Efficacy was higher (75% over 99 weeks) with current ‘Preferred’ cART regimens. There is ongoing loss of efficacy through the second and third years of initial cART, mainly because of participant decision or adverse events. Across all analyses, tenofovir-emtricitabine was more effective than abacavir-lamivudine, and INSTI more effective than NNRTI. As the sensitivity analyses demonstrated, this also applied to the more recent studies of initial cART. Participants with pre-treatment viral load <100,000 copies/mL plasma had significantly better antiviral outcomes on initial cART, a finding persisting within ‘Preferred’ regimens, and similar in magnitude to that favouring February 2013 DHHS ‘Preferred’ over ‘Alternative’ regimens.

Initial cART should aim to induce and maintain long-term virological control. For a disease that presently requires life-long treatment, this analysis demonstrates suboptimal efficacy and durability even with currently ‘Preferred’ initial regimens, corroborating similar findings of the Antiretroviral Therapy Cohort Collaboration [13]. In practice, individuals failing one regimen are likely to be switched to a different, effective combination. Therefore, the present data reflect success or failure of initial cART only, not the absolute failure of all treatment, which we have not examined. Nevertheless, our data show for the first time that almost 15% of participants failed cART between weeks 48 and 144 of therapy. If this trend were ongoing, the majority of initial regimens would fail within 10 years of commencement. As the pace of new antiretroviral development slows, treatment options may become limited as patients are progressively exposed to switching of cART regimens, even in resource-rich settings [14].

Participant decision was the most common identifiable cause of failure, ahead of adverse events or virological failure. This was the case both for studies of ‘Preferred’ regimens, and more recent studies (post-2005). The underlying reasons for this decision, or whether participants re-started cART outside a study, were not routinely reported. Uncovering and systematically documenting reasons for premature cART cessation and subsequent outcomes will be necessary if participants are to be retained more successfully on treatment. Industry-sponsored studies were associated with lower rates of premature cessation due to participant decision or adverse events. It is possible that the cost-free medication and more frequent clinical follow-up commonly mandated within such studies may have attenuated cessation rates. Treatment cessation due to virological failure was consistently low. This was the case even in the earliest cART studies, possibly because the limited treatment options available at the outset of cART prevented drug switches even in the event of study-defined virological failure.

Most treatment failure/cessation occurred early, within the first 48 weeks, with incremental declines of <10% per year at weeks 96 and 144. It may seem self-evident that the longer an individual remains on a single regimen the lower the likelihood of failure, but this may explain why at week 96, only study design characteristics were associated with efficacy, rather than treatment or disease characteristics. There were, however, considerably fewer study groups with data for 96 weeks of follow-up.

Unsurprisingly, of the intention-to-treat strategies used to calculate efficacy, the ‘missing equals failure’ algorithm was associated with greater treatment success, as it has the fewest imputations regarding failure. This raises the possibility that overall efficacy may have been even lower if other, more stringent algorithms had been used exclusively. However, the ‘missing equals failure’ approach arguably more closely reflects actual clinical practice.

The four key international guidelines use the CD4 lymphocyte count as a key marker for when to start cART, with none recommending pre-treatment viral load as an indication [8], [9], [15]. Viral load was removed from DHHS recommendations in 2007, because of data indicating that risk of AIDS or death in individuals receiving cART with pre-treatment CD4 counts ≥350 cells/µL was <2% regardless of viral load [16]. Our findings that: (1) the high-vs.-low viral load and ‘Preferred’-vs.-‘Alternative’ regimen efficacy differences were similar (8.4% vs. 10%, respectively); and (2) ‘Preferred’ regimens had greater efficacy at pre-treatment viral loads <100,000 copies/mL, are therefore striking, since it suggests that the pre-treatment viral load exerts an independent effect upon efficacy, even as guidelines place a primary emphasis on regimen selection. An implication of this finding is that the presence or absence of pre-treatment viral loads ≥100,000 copies/mL may influence the efficacy ultimately reported in studies. In particular, it may help explain the lesser efficacy of abacavir-lamivudine compared to tenofovir-emtricitabine – a finding noted in the ACTG A5202 study [17].

In the existing pre-planned multivariable analyses, neither median viral load, nor the proportion of participants with a pre-treatment viral load ≥ or <100,000 copies/mL was significantly associated with efficacy in the overall multivariable analysis. Within the pre-planned subgroup analyses, there were also no significant interactions between: (1) the regimen type and pre-treatment viral load strata; or (2) the viral load-stratified efficacy and treatment characteristics. However, a post-hoc analysis of our data comparing tenofovir-emtricitabine with abacavir-lamivudine mirrors the gaps in efficacy seen between ‘Preferred’ and ‘Alternative’ regimens (**Table S1 in Appendix S1: Supplementary post-hoc stratified analyses**). At viral loads ≥100,000 copies/mL, overall efficacy with tenofovir-emtricitabine was higher than abacavir-lamivudine (71% [SD 10] vs. 59% [SD 12], respectively; difference 11%; 95%CI 3.6 to 17.7; *p* = 0.004), with a similar difference at viral loads <100,000 copies/mL (79% [SD 12] vs. 67% [SD 14], respectively; difference 14%; 95%CI 6.5 to 20.8; *p*<0.001). The mean follow-up periods were 83 (SD 37) and 74 (SD 38) weeks, respectively. The descriptive findings, both pre-planned and post-hoc, suggest that the overall superior efficacy of tenofovir-emtricitabine (and ‘Preferred’ regimens) is independent of the plasma viral load and further implies that for a given drug or combination, efficacy at lower viral loads is better than at higher viral loads. Not only does this validate current ‘Preferred’ regimens, it argues for future guidelines recommending cART initiation when the plasma viral load rises towards 100,000 copies/mL. Comparable results have been reported recently, albeit limited to 48 weeks’ follow-up [18]. The ACTG A5202 study showed a higher risk of study-defined *virological* failure with abacavir-lamivudine for viral loads ≥100,000 copies/mL at interim analysis (resulting in the unblinding of that stratum), rather than all-cause, intention-to-treat failure. As we did not have premature cessation data stratified by pre-treatment viral load, the results are not directly comparable.

While viral load and regimen type did not significantly interact to influence efficacy, precluding multivariable analyses of these subgroups, it is worth noting that the high-low threshold of 100,000 copies/mL (log_10_5.0) reported in studies is arbitrary. A meta-analysis of efficacy data from individual participants may reveal a clinically relevant association between gradations of viral load and long-term efficacy.

The superiority of tenofovir-emtricitabine over abacavir-lamivudine, although statistically significant on primary analysis, remains confounded by one major issue – that of abacavir-related hypersensitivity. The association between HLA-B*5701 and abacavir-related hypersensitivity, first reported in 2002, is well-described [19], [20]. It would have been advantageous to have more efficacy data with pre-treatment HLA-B*5701 screening. However, due to limited availability, testing for HLA-B*5701 did not become the standard of care until its inclusion in DHHS guidelines from 2007 [21]. By that point, 24 of the 26 groups using abacavir in our review had already commenced, leaving only two studies (total 227 participants) which utilised HLA-B*5701 screening (efficacy 55% [SD 13] over 96 weeks).

Frequency of abacavir-related hypersensitivity is estimated at between 2% and 9%, with some ethnic variation [22]. A meta-analysis of 5,332 patients exposed to abacavir reported a mean incidence of 4% (range 3% to 6%) [23]. Hypersensitivity does contribute to the lesser efficacy of abacavir-lamivudine vs. tenofovir-emtricitabine in our primary analysis, but within the limitations of the source data its relative contribution to higher treatment failure cannot be quantified. Given the insoluble nature of the missing data, one approach to addressing this problem is to infer that the adjusted efficacy difference of 10% between tenofovir-emtricitabine and abacavir-lamivudine is partially due to hypersensitivity (assuming a mean incidence of 4%), with other factors responsible for the balance. For instance, amongst post-2005 studies, the approval of single-tablet, fixed-dose preparations is a possible reason for patients electing to switch away from abacavir mid-clinical trial. During the period of analysis (up to 2010), all such preparations contained tenofovir-emtricitabine as the NRTI backbone. The pairing of tenofovir with emtricitabine, rather than lamivudine, may also contribute to the efficacy difference. Although emtricitabine and lamivudine have closely related chemical structures [24], [25], and are regarded as interchangeable by the DHHS and the World Health Organization [8], [26], emtricitabine has a longer intracellular half-life than lamivudine [27], [28].

The alternative approach is a separate, post-hoc, multivariable analysis excluding the 24 abacavir-lamivudine groups (5,289 participants) that did not use HLA-B*5701 screening. Such an analysis was performed (**Table S2 in Appendix S1: Supplementary post-hoc stratified analyses**); in this model, the comparative difference in efficacy (vs. tenofovir-emtricitabine) was non-significant (coefficient −14.4%; 95%CI −30.2 to 2.5; *p* = 0.175), although the large negative coefficient favoured tenofovir-emtricitabine. In one of the two included abacavir groups (from study CNA109586), hypersensitivity accounted for more premature cessation in the abacavir arm than the comparator tenofovir-emtricitabine arm (6% vs. <1%, respectively), despite HLA-B*5701 screening [29]. With only the two HLA-B*5701-screened groups included, this post-hoc analysis is under-powered to compare abacavir-lamivudine with other NRTI backbones. However, as all other groups were left unchanged from the original primary analysis, the other predictive associations (third drug class, intention-to-treat analysis method) were preserved.

The disadvantage of removing the majority of abacavir-lamivudine groups is that it also removes a large amount of data for the concomitant drug classes from our study. If further studies were then to be excluded on the basis either of currently non-recommended regimens or a non-standard practice, it would also necessitate the exclusion of older cART regimens (e.g. zidovudine-lamivudine), and similarly, the many earlier studies commenced before pre-treatment resistance genotyping became the recommended standard of care in 2006 [30]. With the removal of such a large number of groups from the analysis, the benefits of ecological analysis may be lost, and with it, several comparisons between NRTI backbones and third drug classes.

This systematic review only evaluated entire third drug classes. While this may limit the specificity of the comparisons, assessing individual drugs will result in a loss of analytical power. Our approach is vindicated by long-term prospective data. The adjusted efficacy difference between INSTI and NNRTI classes is consistent with the 10% lower efficacy of efavirenz vs. raltegravir (both ‘Preferred’ agents), as well as with recently-published five-year outcomes of the 004 and STARTMRK studies, and the short-term results of the SPRING-1 study comparing efavirenz with dolutegravir [37], [38], [39]. Examining pooled data can effectively portend long-term prospective results, displaying the value of systematic review.

The favourable efficacy of INSTI- over NNRTI-based initial cART has been noted elsewhere [31]. Our analysis supports this finding, but extends it to include a treatment advantage over boosted PI therapy, and updates earlier meta-analyses conducted prior to INSTI availability. In order to investigate whether the efficacy results for INSTI-based regimens were inflated by the presence of short-term efficacy data for elvitegravir/cobicistat and dolutegravir, a stratified post-hoc analysis was performed separating raltegravir (5 groups, 1,246 participants) from the other INSTI drugs (4 groups, 904 participants). No studies from the primary analysis were excluded. Efficacy overall and at weeks 49 and 96 were similar for raltegravir and non-raltegravir INSTIs (**Table S3 in Appendix S1: Supplementary post-hoc stratified analyses**), although follow-up was shorter for the latter (83 weeks [SD 51] vs. 56 weeks [SD 21]). When multivariable analysis was performed using raltegravir and non-raltegravir INSTIs as separate categorical variables, the results were similar to those of the primary analysis (**Table S4 in Appendix S1: Supplementary post-hoc stratified analyses**). Both raltegravir (coefficient 10.9%; 95%CI 1.2 to 20.6; *p* = 0.028) and non-raltegravir INSTIs (coefficient 13.0%; 95%CI 2.6 to 23.4; *p* = 0.014) were superior third drug options compared to the reference (NNRTIs). These results imply similar efficacy within the INSTI class, a position since adopted by the DHHS in October 2013, when all three INSTIs became listed as ‘Preferred’ options [32].

In the primary analysis, unboosted PIs (regarded as a separate third drug class) were inferior to NNRTIs, while boosted PIs and NNRTIs were similar. The DHHS guidelines do not sanction the use of any unboosted PI, with the exception of unboosted atazanavir, which was used by five groups (932 participants) in our analysis. Unboosted atazanavir was reported as non-inferior when compared to efavirenz and atazanavir/ritonavir in the AI424-034 and ARIES studies, respectively [33], [34]. However, intention-to-treat efficacy in AI424-034 was low (32% at 48 weeks), while the ARIES study used 36 weeks of atazanavir/ritonavir as an induction therapy prior to switching to unboosted atazanavir. Thus whilst atazanavir/ritonavir is a DHHS ‘Preferred’ third drug, unboosted atazanavir is listed as a ‘less satisfactory’ treatment option, one that is neither ‘Preferred’ nor ‘Alternative’ [1]. We conducted a post-hoc analysis considering unboosted atazanavir as a separate third drug class. The results of this analysis were similar to the primary analysis (**Table S5 in Appendix S1: Supplementary post-hoc stratified analyses**). Unboosted atazanavir remained significantly associated with lesser efficacy, as did the other unboosted PIs. The stratified descriptive data supports this; compared to the overall efficacy of unboosted PIs in the primary analysis (42% [SD 11]), the efficacy of unboosted atazanavir and other unboosted PIs were similar −40% (SD 12) and 42% (SD 11), respectively (**Table S6 in Appendix S1: Supplementary post-hoc stratified analyses**). In contrast, the efficacy of atazanavir/ritonavir-based regimens was 72% (SD 8). This supports the findings of the ACTG A5175 study, where the unboosted atazanavir arm was stopped early because of inferior efficacy [35].

The pharmacokinetics of unboosted atazanavir is another reason for caution, as its use effectively precludes combination with tenofovir-containing NRTI backbones, as the latter appears to reduce the bioavailability of atazanavir significantly [1], [36]. In the groups examined in this systematic review, unboosted atazanavir was paired with NRTI backbones selected from older, less effective agents (didanosine, stavudine, zidovudine), which may further attenuate its usefulness in clinical practice.

Despite the advent of fixed-dose combinations, neither daily pill count nor doses significantly predicted efficacy, even amongst ‘Preferred’ or ‘Alternative’ regimens. However, such fixed-dose combinations are a relatively recent development (only three groups had a daily pill count of one), so there were insufficient data to address this question adequately. From our results, however, it appears that while a one-pill-per-day regimen is convenient, it is not necessarily superior. This remains an inference, as importantly, 44% of studies (weighted) used placebo controls, inflating the daily pill count.

We did not analyse ‘Preferred’ and ‘Alternative’ cART regimens by guidelines other than of the DHHS (e.g. World Health Organisation, International Antiviral Society – USA, European AIDS Clinical Society). International ART guidelines have all been derived from the same pool of publicly reported data. Such duplicated analyses are likely to show results similar to the ones we obtained for DHHS-specified cART regimens, i.e. that use of a ‘Preferred’ cART regimen (however defined) resulted in higher efficacy than with ‘Alternative’ regimens. Similarly, despite our updated search being restricted to between 2008 and 2012, an additional sensitivity analysis for post-2008 studies was not performed. This would have effectively duplicated the ‘Preferred’-vs.-‘Alternative’ regimen analysis, while arbitrarily excluding studies of currently ‘Preferred’ (and therefore relevant) regimens that commenced pre-2008.

There are several limitations to our study. Chief of these is that the base unit of analysis in our methodology was the treatment group. Hence ours was an ecological analysis, using mean aggregate data, rather than individual participant data. Also, other, more commonly-reported meta-analyses of cART aim to combine similarly-designed, randomised comparisons of efficacy/failure to achieve data homogeneity. Such analyses therefore have a different hypothesis, i.e. they evaluate the comparison, whereas a study such as ours describes overall efficacy and failure in each group exposed to the treatment drug(s). By including a wide variety of treatments and settings, our data set is more heterogeneous; a meta-regression approach permits exploration of potential sources of heterogeneity in the estimation of efficacy. This would not be possible with a ‘typical’ meta-analysis of similar studies. However, because broad associations are identified, it is not possible to infer causality, and it would be incorrect to deduce the impact of a particular cART regimen on efficacy in participants of a certain age, gender, race, location or clinical status. However, as standardised outcome measures (virological efficacy and failure) have been applied to a large weighting of participants, it is best suited to representing the likely outcomes for populations with a similar profile, reporting associations that may advise public policy, like the DHHS and other international antiretroviral guidelines, and identifying topics for future study. As most study groups were of predominantly white race, this may limit applicability to currently resource-limited settings.

A multivariable approach was used to interrogate heterogeneity within the efficacy data. With this approach, bias due to missing data may allow some clinically relevant sources of heterogeneity to be missed whilst other, non-relevant sources might reach statistical significance. The large number of groups examined reduces, but does not eliminate, the risk of such bias. The inability of our study to assess the effect of HLA-B*5701 screening on the relative efficacy of abacavir-lamivudine is a key example of this, and one which necessitated post-hoc analysis in an attempt at characterisation. The failure of some covariates to reach significance may also highlight which data are currently poorly reported in studies. We were able to source some non-published data from pharmaceutical sponsors, but very little from academic sponsors. For each subgroup analysis, population numbers changed, so comparisons between subgroups can only be inferred. For most studies, randomisation was not stratified by pre-treatment viral load. It is likely that variables not reported by viral loads strata (previous AIDS events, CD4 lymphocyte count, adherence, co-infection with viral hepatitis) can partly explain the differences seen between high and low viral load strata. This limitation does not affect other subgroup analyses. Finally, HIV viral load was used as the primary outcome measure, rather than a clinical endpoint.

Our data identifies pre-treatment HIV-1 viral load as a determinant of efficacy, which should prompt a re-examination of its place in treatment guidelines, and also suggests that HIV-infected adults should initiate cART for plasma viral loads rising towards 100,000 copies/mL. Whether this difference is driven by lesser antiviral potency at high viral loads is unknown because of missing data, but requires further investigation. One possible option would be a prospective study comparing triple- and quadruple-drug combinations as initial therapy in high viral loads. The medium-term efficacy of initial cART alone remains unsatisfactory, and prospective studies require longer follow-up and better reporting of adverse events and reasons for participant-initiated treatment cessation. As an ecological analysis of pooled outcomes, our findings are associations rather than causes, best applied to populations with demographics similar to our source data. Future analyses performed using data from individual participants are needed to allow specific causality to be inferred for the trends identified in this study.

## Supporting Information

Appendix S1 **Supplementary post-hoc stratified analyses.**
(DOC)

Checklist S1 **2009 PRISMA Checklist.**
(DOC)

Protocol S1 **Statistical Analysis Plan.**
(PDF)
