# Supplementary material for: Efficacy of Initial Antiretroviral Therapy for HIV-1 Infection in Adults: A Systematic Review and Meta-Analysis of 114 Studies with up to 144 Weeks' Follow-Up
Source: PLoS One. 2014 May 15;9(5):e97482. doi: 10.1371/journal.pone.0097482 (PMC4022522; doi:10.1371/journal.pone.0097482)
Supplement: Appendix S1 — Supplementary post-hoc stratified analyses. (DOC) [file pone.0097482.s001.doc]

**Appendix S1: Supplementary post-hoc stratified analyses**

**Table S1. Efficacy of tenofovir-emtricitabine vs. abacavir-lamivudine: overall and by pre-treatment plasma viral load**

|  | **Overall** | | **Pre-treatment HIV viral load <100,000 copies/mL** | | **Pre-treatment HIV viral load ≥100,000 copies/mL** | |
| --- | --- | --- | --- | --- | --- | --- |
|  | **Tenofovir-emtricitabine** | **Abacavir-lamivudine** | **Tenofovir-emtricitabine** | **Abacavir-lamivudine** | **Tenofovir-emtricitabine** | **Abacavir-lamivudine** |
| **Groups (participants)** | 47 (11,001) | 26 (5,516) | 29 (8,100) | 24 (5,232) | 29 (8,100) | 24 (5,232) |
| **Mean follow-up, weeks (SD)** | 83 (38) | 74 (37) | 83 (37) | 74 (38) | 83 (37) | 74 (38) |
| **Antiviral efficacy, % (SD)** | 72 (9.7) | 63 (6.8) | 79 (12) | 67 (14) | 71 (10) | 59 (12) |
| **Premature treatment cessation, % (SD)^*^** |  |  |  |  |  |  |
| **Participant decision** | 8.1 (5.2) | 11 (4.4) | No data | No data | No data | No data |
| **Adverse events** | 5.7 (3.5) | 8.3 (4.0) | No data | No data | No data | No data |
| **Virological failure** | 3.7 (3.7) | 2.0 (2.7) | No data | No data | No data | No data |
| **Other** | 3.1 (2.4) | 4.2 (2.2) | No data | No data | No data | No data |
| **Total** | 20 (8.2) | 26 (8.6) | No data | No data | No data | No data |

* Data for premature cessation stratified by pre-treatment viral load not available.

SD, standard deviation.

**Table S2. NRTI associations^*^ with overall efficacy of initial antiretroviral therapy: HLA-B*5701-unscreened studies excluded**

|  |  |  | **Univariable Analysis** | | | | | **Multivariable Analysis^†^** | | | |
| --- | --- | --- | --- | --- | --- | --- | --- | --- | --- | --- | --- |
|  |  | **Efficacy, % (SD)** | **Coefficient, %^‡^** | **95%CI** | ***p*** | ***p* trend** | ***r*^2^ (%)** | **Coefficient, %^‡^** | **95%CI** | ***p*** | ***p* group** |
| **NRTI backbone** | **TDF-FTC** | 73 (10) | Ref |  |  |  |  | Ref |  |  |  |
|  | **AZT-3TC** | 48 (15) | -19.3 | -24.3, -14.3 | <0.001 |  |  | -9.2 | -15.0, -3.2 | 0.002 |  |
|  | **d4T-3TC** | 55 (12) | -14.7 | -20.9, -8.5 | <0.001 |  |  | -7.1 | -13.7, -0.6 | 0.031 |  |
|  | **ABC-3TC^¶^** | 55 (13) | -10.0 | -28.8, 8.8 | 0.295 |  |  | -14.4 | -30.2, 2.5 | 0.175 |  |
|  | **d4T-ddI** | 44 (13) | -25.0 | -31.9, -18.1 | <0.001 |  |  | -14.1 | -21.6, -6.5 | <0.001 |  |
|  | **Nil/2 NRTIs** | 66 (15) | -5.8 | -12.6, 0.9 | 0.088 |  |  | -0.5 | -6.7, 5.8 | 0.885 |  |
|  | **TDF-3TC** | 69 (4.6) | -1.0 | -11.3, 9.2 | 0.842 |  |  | 1.4 | -7.1, 10.0 | 0.740 |  |
|  | **ddI-3TC** | 65 (8.4) | -8.0 | -18.1, 2.1 | 0.119 |  |  | -3.6 | -13.3, 6.1 | 0.465 |  |
|  | **AZT-ddI** | 42 (3.5) | -30.2 | -49.6, -10.7 | 0.003 |  |  | -31.5 | -48.8, -14.1 | <0.001 |  |
|  | **ddI-FTC** | 78 (-) | 5.4 | -19.1, 30.0 | 0.664 |  |  | 13.7 | -6.6, 34.0 | 0.183 |  |
|  | **3TC** | 67 (-) | -5.6 | -33.9, 22.7 | 0.696 | <0.001 | 35.3 | -14.7 | -39.4, 10.0 | 0.241 | 0.001 |

* NRTI backbone, third drug class and the intention-to-treat analysis method were all significantly associated with efficacy in this post-hoc multivariable analysis. Only the NRTI backbone associations are shown. Study phase, genotype/CD4 eligibility restrictions, pills and doses per day were significant on univariable analysis but not multivariable. Co-infection with hepatitis B or C were excluded from the multivariable analysis because >20% of groups were missing data.

† Study sponsorship, placebo use, country/region of recruitment, haemoglobin/viral load/liver function eligibility restrictions, race, risk factors for HIV infection, sex, previous AIDS events, pre-treatment viral load/CD4 count, dosing relative to food, serious adverse events, and clinical/laboratory adverse events of at least moderate severity were variables not significant on univariable analysis.

‡ Coefficient represents the adjusted percentage difference in outcomes relative to a unit increase in any variable (or relative to the reference variable within a category).

¶ Includes only the two abacavir-lamivudine groups utilising pre-treatment HLA-B*5701 screening; 24 abacavir-lamivudine groups excluded.

SD, standard deviation; CI, confidence interval; TDF, tenofovir; FTC, emtricitabine; AZT, zidovudine; 3TC, lamivudine; d4T, stavudine; ddI, didanosine; ABC, abacavir; ELV, elvucitabine; NRTI, nucleoside reverse transcriptase inhibitor.

**Table S3. Efficacy and premature treatment cessation in raltegravir and other INSTI drugs**

|  | **Overall** | | **48 weeks** | | **96 weeks** | |
| --- | --- | --- | --- | --- | --- | --- |
|  | **Raltegravir** | **Other INSTI^*^** | **Raltegravir** | **Other INSTI^*^** | **Raltegravir** | **Other INSTI^*^** |
| **Groups (participants)** | 5 (1,246) | 4 (904) | 5 (1,246) | 4 (904) | 3 (476) | 1 (155) |
| **Mean follow-up, weeks (SD)** | 83 (51) | 56 (21) | 48 | 48 | 96 | 96 |
| **Antiviral efficacy, % (SD)** | 82 (6.0) | 85 (1.8) | 86 (2.8) | 87 (2.0) | 81 (1.9) | 82 (-) |
| **Premature treatment cessation, % (SD)^†^** |  |  |  |  |  |  |
| **Participant decision** | 4.5 (2.4) | 4.7 (0.7) | 3.0 (1.3) | 4.0 (1.7) | 3.4 (3.5) | 5 (-) |
| **Adverse events** | 2.0 (1.4) | 2.9 (1.4) | 1.5 (1.0) | 2.9 (1.4) | 3.3 (1.1) | 1 (-) |
| **Virological failure** | 2.8 (1.8) | 0.8 (0.5) | 2.2 (2.1) | 0.8 (0.5) | 2.1 (0.3) | 0 (-) |
| **Other** | 3.5 (4.0) | 1.8 (1.3) | 1.6 (1.4) | 1.7 (0.9) | 4.9 (2.0) | 4 (-) |
| **Total** | 13 (6.8) | 10 (1.3) | 8.3 (2.3) | 9.3 (2.6) | 14 (1.3) | 10 (-) |

* Other INSTI: dolutegravir, elvitegravir/cobicistat.

† Data for premature cessation stratified by pre-treatment viral load not available.

INSTI, integrase strand transfer inhibitor; SD, standard deviation.

**Table S4. Third drug class associations* with overall efficacy of initial antiretroviral therapy: raltegravir isolated as a separate category**

|  |  |  | **Univariable Analysis** | | | | | **Multivariable Analysis^†^** | | | |
| --- | --- | --- | --- | --- | --- | --- | --- | --- | --- | --- | --- |
|  |  | **Efficacy, % (SD)** | **Coefficient, %^‡^** | **95%CI** | ***p*** | ***p* trend** | ***r*^2^ (%)** | **Coefficient, %^‡^** | **95%CI** | ***p*** | ***p* group** |
| **Third drug class** | **NNRTI** | 61 (16) | Ref |  |  |  |  | Ref |  |  |  |
|  | **PI (boosted)** | 68 (9.1) | 2.9 | -1.0, 6.8 | 0.140 |  |  | -0.9 | -4.7, 2.9 | 0.644 |  |
|  | **PI (unboosted)** | 41 (11) | -19.2 | -23.7, -14.8 | <0.001 |  |  | -15.1 | -19.5, -10.6 | <0.001 |  |
|  | **NRTI/2 NRTIs** | 50 (12) | -13.3 | -20.4, -6.3 | <0.001 |  |  | -10.8 | -17.5, -4.1 | 0.002 |  |
|  | **Raltegravir** | 82 (6.0) | 16.9 | 6.6, 27.2 | 0.001 |  |  | 10.9 | 1.2, 20.6 | 0.028 |  |
|  | **Other INSTI^¶^** | 85 (1.8) | 20.4 | 9.0, 31.7 | <0.001 |  |  | 13.0 | 2.6, 23.4 | 0.014 |  |
|  | **INSTI+PI** | 64 (3.6) | 0.9 | -13.3, 15.2 | 0.897 |  |  | -6.8 | -20.7, 7.1 | 0.335 |  |
|  | **NNRTI+PI** | 40 (16) | -21.2 | -36.8, -5.6 | 0.008 |  |  | -27.2 | -41.9, -12.5 | <0.001 |  |
|  | **CCR5/CCR5+PI** | 60 (4.7) | -0.9 | -17.4, 15.5 | 0.911 | <0.001 | 42.7 | -2.6 | -17.5, 12.3 | 0.729 | <0.001 |

* Third drug class, NRTI backbone and the intention-to-treat analysis method were all significantly associated with efficacy in this post-hoc multivariable analysis. Only the third drug class associations are shown. Study phase, genotype/CD4 eligibility restrictions, pills and doses per day were significant on univariable analysis but not multivariable. Co-infection with hepatitis B or C were excluded from the multivariable analysis because >20% of groups were missing data.

† Study sponsorship, placebo use, country/region of recruitment, haemoglobin/viral load/liver function eligibility restrictions, race, risk factors for HIV infection, sex, previous AIDS events, pre-treatment viral load/CD4 count, dosing relative to food, serious adverse events, and clinical/laboratory adverse events of at least moderate severity were variables not significant on univariable analysis.

‡ Coefficient represents the adjusted percentage difference in outcomes relative to a unit increase in any variable (or relative to the reference variable within a category).

¶ Other INSTI: dolutegravir, elvitegravir/cobicistat.

SD, standard deviation; CI, confidence interval; NRTI, nucleoside reverse transcriptase inhibitor; NNRTI, non-nucleoside reverse transcriptase inhibitor; PI, protease inhibitor; INSTI, integrase strand transfer inhibitor; CCR5, chemokine receptor 5 inhibitor.

**Table S5. Third drug class associations^*^ with overall efficacy of initial antiretroviral therapy: unboosted atazanavir isolated as a separate category**

|  |  |  | **Univariable Analysis** | | | | | **Multivariable Analysis^†^** | | | |
| --- | --- | --- | --- | --- | --- | --- | --- | --- | --- | --- | --- |
|  |  | **Efficacy, % (SD)** | **Coefficient, %^‡^** | **95%CI** | ***p*** | ***p* trend** | ***r*^2^ (%)** | **Coefficient, %^‡^** | **95%CI** | ***p*** | ***p* group** |
| **Third drug class** | **NNRTI** | 61 (16) | Ref |  |  |  |  | Ref |  |  |  |
|  | **PI (boosted)** | 68 (9.1) | 2.9 | -1.0, 6.8 | 0.141 |  |  | -0.9 | -4.7, 2.9 | 0.644 |  |
|  | **Unboosted atazanavir** | 40 (12) | -20.9 | -31.3, -10.5 | <0.001 |  |  |  |  |  |  |
|  | **Other unboosted PI^¶^** | 42 (SD 11) | -19.0 | -23.7, -14.2 | <0.001 |  |  | -15.1 | -19.5, -10.6 | <0.001 |  |
|  | **NRTI/2 NRTIs** | 50 (12) | -13.3 | -20.4, -6.2 | <0.001 |  |  | -10.8 | -17.5, -4.1 | 0.002 |  |
|  | **INSTI** | 84 (4.7) | 18.5 | 10.7, 26.2 | <0.001 |  |  | 13.0 | 2.6, 23.4 | 0.014 |  |
|  | **INSTI+PI** | 64 (3.6) | 0.9 | -13.4, 15.2 | 0.897 |  |  | -6.8 | -20.7, 7.1 | 0.335 |  |
|  | **NNRTI+PI** | 40 (16) | -21.2 | -36.9, -5.5 | 0.008 |  |  | -27.2 | -41.9, -12.5 | <0.001 |  |
|  | **CCR5/CCR5+PI** | 60 (4.7) | -0.9 | -17.4, 15.5 | 0.911 | <0.001 | 42.7 | -2.6 | -17.5, 12.3 | 0.729 | <0.001 |

* Third drug class, NRTI backbone and the intention-to-treat analysis method were all significantly associated with efficacy in this post-hoc multivariable analysis. Only the third drug class associations are shown. Study phase, genotype/CD4 eligibility restrictions, pills and doses per day were significant on univariable analysis but not multivariable. Co-infection with hepatitis B or C were excluded from the multivariable analysis because >20% of groups were missing data.

† Study sponsorship, placebo use, country/region of recruitment, haemoglobin/viral load/liver function eligibility restrictions, race, risk factors for HIV infection, sex, previous AIDS events, pre-treatment viral load/CD4 count, dosing relative to food, serious adverse events, and clinical/laboratory adverse events of at least moderate severity were variables not significant on univariable analysis.

‡ Coefficient represents the adjusted percentage difference in outcomes relative to a unit increase in any variable (or relative to the reference variable within a category).

¶ Other unboosted PI: amprenavir, fosamprenavir, indinavir, nelfinavir, ritonavir.

SD, standard deviation; CI, confidence interval; NRTI, nucleoside reverse transcriptase inhibitor; NNRTI, non-nucleoside reverse transcriptase inhibitor; PI, protease inhibitor; INSTI, integrase strand transfer inhibitor; CCR5, chemokine receptor 5 inhibitor.

**Table S6. Efficacy and premature treatment cessation within the unboosted PI class: compared to atazanavir/ritonavir**

|  | **Unboosted atazanavir** | **Other unboosted PI^*^** | **Atazanavir/ritonavir** |
| --- | --- | --- | --- |
| **Groups (participants)** | 5 (932) | 33 (4,754) | 12 (2,445) |
| **Mean follow-up, weeks (SD)** | 53 (17) | 65 (31) | 96 (44) |
| **Antiviral efficacy, % (SD)** | 40 (12) | 42 (11) | 72 (8.0) |
| **Premature treatment cessation, % (SD)^†^** |  |  |  |
| **Participant decision** | 6.0 (2.2) | 14 (5.6) | 10 (5.7) |
| **Adverse events** | 5.6 (1.7) | 9.9 (7.6) | 5.7 (2.9) |
| **Virological failure** | 2.6 (2.4) | 3.4 (3.5) | 1.9 (1.9) |
| **Other** | 3.3 (1.0) | 4.9 (4.3) | 2.7 (2.1) |
| **Total** | 17 (5.5) | 31 (11) | 20 (8.6) |

* Other unboosted PI: amprenavir, fosamprenavir, indinavir, nelfinavir, ritonavir.

† Data for premature cessation stratified by pre-treatment viral load not available.

PI, protease inhibitor; SD, standard deviation.
