# Supplementary material for: Efficacy of Initial Antiretroviral Therapy for HIV-1 Infection in Adults: A Systematic Review and Meta-Analysis of 114 Studies with up to 144 Weeks' Follow-Up
Source: PLoS One. 2014 May 15;9(5):e97482. doi: 10.1371/journal.pone.0097482 (PMC4022522; doi:10.1371/journal.pone.0097482)
Supplement: Protocol S1 — Statistical Analysis Plan. (PDF) [file pone.0097482.s003.pdf]

Protocol S1: Statistical Analysis Plan

Factors influencing the efficacy of initial anti-retroviral therapy: a meta-analysis of 40,124 HIV-1 infected adults with up to 144 weeks' follow-up

Frederick J. Lee
Janaki Amin
Andrew Carr

Design

This study is a systematic review and meta-analysis of prospective studies of initial combination anti-retroviral therapy (cART) published and/or reported up to December 2012.

Objectives

Primary

The primary outcome is to describe the overall antiviral efficacy of initial cART. Antiviral efficacy is defined as the proportion of participants in a single treatment group with an undetectable (study-defined) plasma HIV-1 viral load at follow-up, as reported by an intent-to-treat analysis method.

Secondary

- Identify components of: year of study commencement, study design, eligibility criteria, participant and disease characteristics and treatment components and parameters that are independently associated with/predictors of efficacy of initial cART (see **Statistical Methods** below for a complete list).
- Objectives by duration of follow-up:
    - efficacy of initial cART at the following time points following commencement (week 0):
        - through week 48;
        - through week 96;
        - through week 144.
    - identify components of: year of study commencement, study design, eligibility criteria, participant and disease characteristics and treatment components and parameters that are independently associated with/predictors of efficacy of initial cART:
        - through week 48;
        - through week 96;
        - through week 144.
    - describe the change in efficacy of initial cART:
        - between weeks 48 and 96;
        - between weeks 96 and 144.
    - identify components of: year of study commencement, study design, eligibility criteria, participant and disease characteristics and treatment components and parameters that are independently associated with/predictors of efficacy of initial cART:
        - between Weeks 48 and 96;
        - between Weeks 96 and 144.
- Objectives by pre-treatment HIV-1 plasma viral load:
    - efficacy of initial cART in participants with a pre-treatment HIV-1 plasma viral load of:
        - ≥100,000 copies/mL;
        - <100,000 copies/mL.
    - identify components of: year of study commencement, study design, eligibility criteria, participant and disease characteristics and treatment components and parameters that are

independently associated with/predictors of efficacy of initial cART when the pre-treatment HIV-1 plasma viral load is:
    - ≥100,000 copies/mL;
    - <100,000 copies/mL.

- Objectives according to United States Department of Health and Human Services (DHHS)-defined regimen type, as specified by the 2012 DHHS Guidelines for the Use of Antiretroviral Agents in HIV-1-Infected Adults and Adolescents [1]:
    - efficacy of initial cART when the treatment is:
        - a 2012 DHHS-‘Preferred’ regimen;
        - a 2012 DHHS-‘Alternative’ regimen;
    - Identify components of: year of study commencement, study design, eligibility criteria, participant and disease characteristics and treatment components and parameters that are independently associated with/predictors of efficacy of initial cART when the treatment is:
        - a 2012 DHHS-‘Preferred’ regimen;
        - a 2012 DHHS-‘Alternative’ regimen;

Other outcomes

- Premature cessation of initial cART:
    - the proportion of participants prematurely ceasing initial cART:
        - overall;
        - by Week 48;
        - by Week 96;
        - by Week 144.
    - reasons for premature cessation of initial cART, expressed as one of the following categories:
        - adverse events;
        - participant decision (voluntary withdrawal or loss to follow-up);
        - virological failure:
        - all other reasons.
    - identify components of: year of study commencement, study design, eligibility criteria, participant and disease characteristics and treatment components and parameters that are independently associated with/predictors of premature cessation:
        - total premature cessation;
        - premature cessation due to adverse events;
        - premature cessation due to participant decision;
        - premature cessation due to virological failure;
        - premature cessation due to all other reasons.
- Overall absolute rise in CD4 count (expressed as cells/mm^3) of participants, by on-treatment analysis (not intent-to-treat).

Selection of studies

Inclusion criteria

- Conducted in consenting adult participants (aged ≥18 years) who were treatment-naïve at time of enrolment into study.
- Prospective study design, either randomised or cohort.
- Reported efficacy as proportion of participants with an undetectable (study-defined) HIV-1 plasma viral load, by an intent-to-treat analysis method.
- Minimum follow-up duration of 48 weeks.

Exclusion criteria

- Retrospective or cross-sectional study design.
- Studies of cART regimens listed as 'not acceptable' due to toxicity concerns in key treatment guidelines up to 2012
  - regimens previously, but not currently, recommended by key international treatment guidelines are included, as are novel regimens used in pilot studies without toxicity concerns (at the discretion of the investigators).
- Studies of multiple or variable cART regimens within a single treatment arm
  - cross-over and cluster-randomised trials are excluded;
  - treatment arms with fixed but unspecified dual nucleoside analogue reverse transcriptase inhibitor (NRTI) backbones and a common third drug are allowed for inclusion.
- Studies of directly-observed therapy.
- Studies reporting data for <48 weeks of cART.

Data sources and search strategy

Studies published and/or reported between 2008 and 2012

The search period extends from January 1, 2008 to December 31, 2012. No language restriction will be applied.

The following electronic databases will be searched:

- MEDLINE, via the PubMed search engine (http://www.ncbi.nlm.nih.gov/pubmed/);
- the Cochrane Central Register of Controlled Trials (http://onlinelibrary.wiley.com/o/cochrane/cochrane_clcentral_articles_fs.html/);
- United States National Institutes of Health clinical trials registry (http://www.clinicaltrials.gov/);
- International Standard Randomised Controlled Trial Numbers (ISRCTN) registry (http://www.controlled-trials.com/).

For each database, the search strategy will be:

- '["drug"] AND (HIV OR anti-retroviral) AND (cohort OR randomised trial)'
  - "drug" = generic (including any pre-approval code names) of an antiretroviral drug.

The abstracts of the following key scientific meetings between 2008 and 2012 will be searched:

- Conference on Retroviruses and Opportunistic Infections (http://www.retroconference.org);
- International AIDS Society Conference (http://www.iasociety.org/);
- Interscience Conference on Antimicrobial Agents and Chemotherapy (http://www.icaac.org/);
- International Congress on Drug Therapy in HIV.

Studies published and/or reported pre-2008

Studies from the authors' earlier meta-analysis will be combined with 2008-2012 results for a final pool of eligible studies of initial cART [2]. All pre-2008 studies will be manually reviewed by one author (FL), in duplicate, for eligibility, with any discrepancy to be discussed with a second author (AC).

Additional data sources

Drug labels and medical reviews of individual antiretroviral drugs published by regulatory bodies:

- United States Food and Drug Administration (http://www.fda.gov/);
- European Medicines Agency (http://www.ema.europa.au/).

Study synopses released into the public domain by pharmaceutical companies:

- Abbott (http://www.abbott.com/);

- Bristol Myers-Squibb (http://www.bms.com/);
- Gilead Sciences (http://www.gilead.com/);
- GlaxoSmithKline (http://www.gsk-clinicalstudyregister.com/);
- Roche (http://www.roche-trials.com/).

Data extraction and management

Study arms will be regarded as individual treatment groups (i.e. a randomised study of three treatment regimens will be counted as three treatment groups), ensuring that the same participants are not included twice in the meta-analysis. A single data collection form will be generated and used to extract information from all the included studies. Data from all the studies will then be entered into two duplicate databases by one author (FL). Each database will be reviewed by direct comparison for any discrepancies, which will be resolved by referencing the primary source, and then vetted by a second review author (AC).

In cases where no data is reported for a particular variable, the study sponsors and/or authors will be approached in the first instance for any unreported data. If no such data is available, then it will be entered as being missing/unavailable, so that there are no blank spaces in the database. There will be no imputations for missing data.

Statistical methods

All analysis will be performed using STATA® software version 11 (StataCorp LP, College Station, Texas, USA), with the meta-analysis package.

Summary measures and results synthesis

The unit of analysis will be the treatment group. The primary summary measure is the proportion of participants within a group expressing any given variable (as a percentage), and the mean (for continuous variables, including proportions). For combined data, efficacy outcomes will be calculated as a mean percentage, weighted by the size (i.e. the number of participants in each group).

Demographic and pre-treatment characteristics

The following demographic and pre-treatment characteristics will be collected:

- age
- gender
- race
  - ‘white’ (including individuals of Hispanic origin);
  - ‘black’ (refers to any black participants, regardless of geographic region);
  - ‘other’ (refers to any non-white/non-black populations, including indigenous populations).
- risk factors for HIV acquisition
  - male sex with males (MSM);
  - injecting drug use (IDU);
  - heterosexual;
  - other.
- previous AIDS/CDC Category C event
- pre-treatment CD4 count and HIV-1 plasma viral load
- pre-treatment evidence of hepatitis C or hepatitis B virus co-infection
- pre-treatment body weight

Identification of predictor variables

A linear regression approach will be used to identify any variables independently associated with efficacy of initial cART. The analysis will be performed in multivariable linear regression models using backwards, stepwise selection of variables. All variables with a p value of ≤0.05 in univariate analysis will be assessed in building the final multivariable models. If it arises that variables listed both as an eligibility criterion and a pre-treatment characteristic (e.g. criteria for minimum CD4 cell count at study entry and pre-treatment CD4 cell count) were significant in the univariate analysis, only the pre-treatment measure will be assessed in building the final multivariable model. Any variables that have <80% of data available across all treatment groups (i.e. >20% of data for that variable is entered as 'missing') will be excluded from the multivariable model, regardless of whether it is significant on the univariable analysis.

Inclusion of the year of study commencement into the multivariable analysis could bias results towards studies using newer antiretroviral classes, fixed-dose combinations or routine pre-treatment genotyping. Therefore, the multivariable analysis should be performed both with and without the year of study commencement as a predictor variable (if $p \leq 0.05$ on univariable analysis) by way of a sensitivity analysis.

Differences in treatment group sizes will be adjusted for using the standard error of the mean for each group efficacy.

Variables included in each of the secondary outcome linear regression analyses are:

- study design
    - study phase
    - placebo-controlled
    - study sponsorship
    - country/region of recruitment
    - ITT analysis method
- year of commencement
- eligibility criteria
    - pre-treatment haemoglobin
    - pre-treatment hepatic transaminases
    - pre-treatment CD4 cell count
    - pre-treatment HIV-1 plasma viral load
    - pre-treatment resistance genotype
- treatment characteristics
    - NRTI backbone
    - third drug class
    - daily pill count
    - doses per day
    - dosing relative to food
- demographic and pre-treatment characteristics (as listed above)
- adverse events
    - clinical events of ≥grade 2
    - serious adverse events
    - laboratory events of ≥grade 3

Other

Heterogeneity will be assessed using the I^2 statistic [3], while differences between means will be compared using Student's *t*-test.

References

1. Department of Health and Human Services (DHHS) Panel on Antiretroviral Guidelines for Adults and Adolescents. Guidelines for the use of antiretroviral agents in HIV-1-infected adults and adolescents. Rockville, MA, USA: Department of Health and Human Services; 2012 [updated March 27].
2. Carr A, Amin J. Efficacy and tolerability of initial antiretroviral therapy: a systematic review. *AIDS* 2009; **23**: 343-53.
3. Higgins JP, Thompson SG, Deeks JJ, Altman DG. Measuring inconsistency in meta-analyses. *BMJ* 2003; **327**: 557-60.
